# CNS-associated macrophages contribute to intracerebral aneurysm pathophysiology

**DOI:** 10.1186/s40478-024-01756-5

**Published:** 2024-03-18

**Authors:** Martina Glavan, Ana Jelic, Damien Levard, Juhana Frösen, Sara Keränen, Bart A. A. Franx, Ana-Rita Bras, Estelle R. Louet, Ádám Dénes, Mario Merlini, Denis Vivien, Marina Rubio

**Affiliations:** 1 UNICAEN, INSERM U1237, Etablissement Français du Sang, Physiopathology and Imaging of Neurological Disorders (PhIND), Cyceron, Institut Blood and Brain @ Caen-Normandie (BB@C), PHIND Boulevard Henri Becquerel, Normandie University, 14000 Caen Cedex, Caen France; 2 Department of Clinical Research, Caen Normandie University Hospital, Caen, France; 3 Hemorrhagic Brain Pathology Research Group, Kuopio University Hospital and AIV Institute for Molecular Medicine, University of Eastern Finland, Kuopio, Finland; 4 Dept of Neurosurgery, Tampere University Hospital and Hemorrhagic Brain Pathology Research Group, Tampere University, Tampere, Finland; 5 Translational Neuroimaging Group, Center for Image Sciences, University Medical Center Utrecht and Utrecht University, Utrecht, The Netherlands; 6 “Momentum” Laboratory of Neuroimmunology, Institute of Experimental Medicine, Budapest, Hungary; 7 János Szentágothai Doctoral School of Neurosciences, Schools of PhD Studies, Semmelweis University, Budapest, Hungary; 8 Present Address: Department of Neuroscience, Yale School of Medicine, Yale University, 333 Cedar Street, New Haven, CT 06510 USA

**Keywords:** CNS-associated macrophages, Intracerebral aneurysms, Middle cerebral artery, Vascular inflammation, MRI

## Abstract

Intracerebral aneurysms (IAs) are pathological dilatations of cerebral arteries whose rupture leads to subarachnoid hemorrhage, a significant cause of disability and death. Inflammation is recognized as a critical contributor to the formation, growth, and rupture of IAs; however, its precise actors have not yet been fully elucidated. Here, we report CNS-associated macrophages (CAMs), also known as border-associated macrophages, as one of the key players in IA pathogenesis, acting as critical mediators of inflammatory processes related to IA ruptures. Using a new mouse model of middle cerebral artery (MCA) aneurysms we show that CAMs accumulate in the IA walls. This finding was confirmed in a human MCA aneurysm obtained after surgical clipping, together with other pathological characteristics found in the experimental model including morphological changes and inflammatory cell infiltration. In addition, in vivo longitudinal molecular MRI studies revealed vascular inflammation strongly associated with the aneurysm area, i.e., high expression of VCAM-1 and P-selectin adhesion molecules, which precedes and predicts the bleeding extent in the case of IA rupture. Specific CAM depletion by intracerebroventricular injection of clodronate liposomes prior to IA induction reduced IA formation and rupture rate. Moreover, the absence of CAMs ameliorated the outcome severity of IA ruptures resulting in smaller hemorrhages, accompanied by reduced neutrophil infiltration. Our data shed light on the unexplored role of CAMs as main actors orchestrating the progression of IAs towards a rupture-prone state.

**Graphical abstract:**

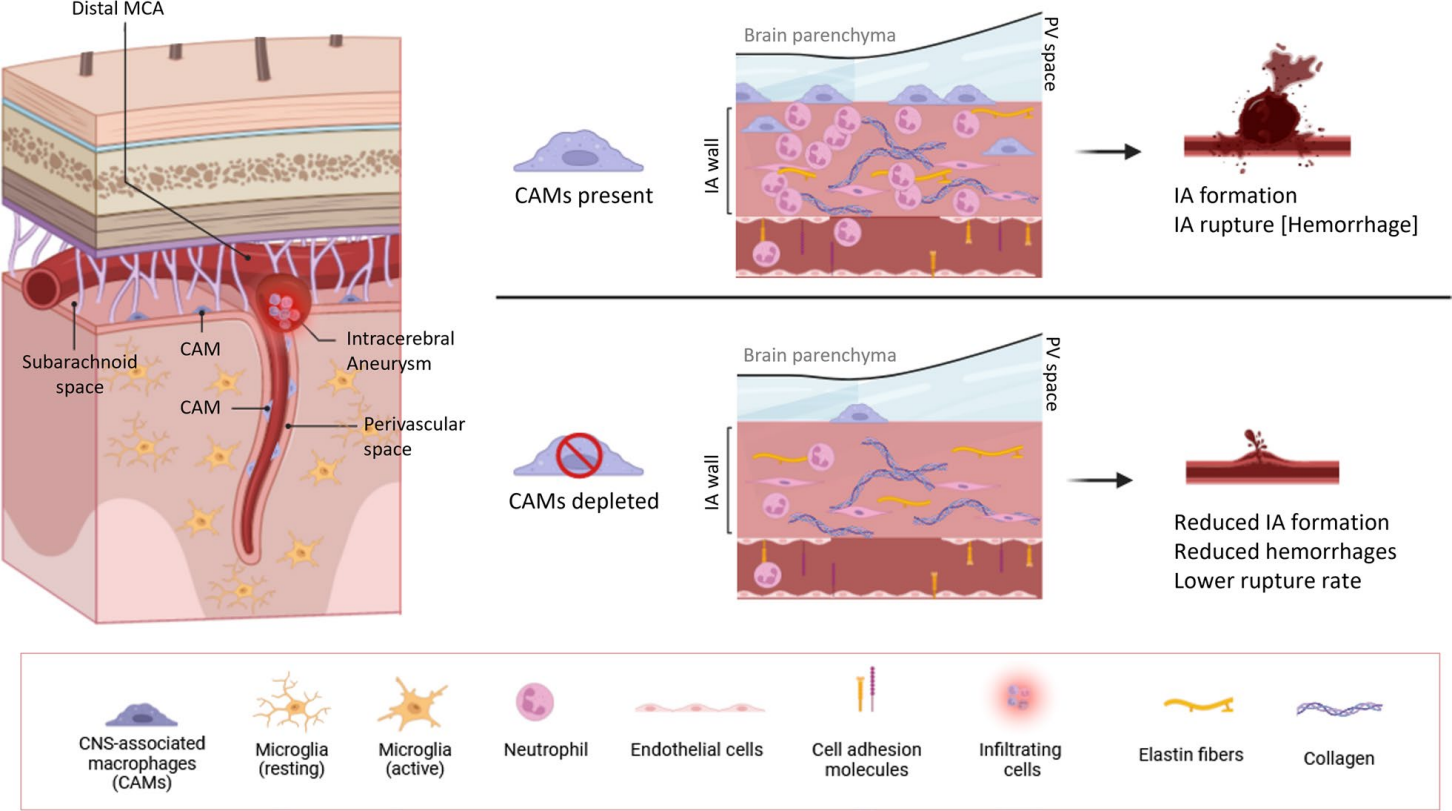

**Supplementary Information:**

The online version contains supplementary material available at 10.1186/s40478-024-01756-5.

## Introduction

Intracerebral aneurysms (IA) are pathological dilatations of the cerebral arteries which may rupture, leading to intracerebral or subarachnoid hemorrhage (SAH) [21]. The SAH mortality rate exceeds 40% [33], and is associated with high morbidity affecting productive life [28]. Thus, a ruptured IA requires prompt medical treatment. Management of unruptured IA is; however, challenging given that both of the currently available preventive treatments, microsurgical clipping and endovascular coiling, have morbidity and mortality risks of up to 5% [19, 36]. Consequently, the treatment benefits must be cautiously balanced against the risks of rupture. Hence, the identification and assessment of prognostic risk factors for rupture are essential for clinical decision-making.

Although numerous studies have shown that inflammation plays a critical role in aneurysm formation, growth, and rupture [3, 16, 46], the specific role of CNS-associated macrophages (CAMs) (also referred to as border-associated macrophages or BAMs) has not yet been studied in this context. CAMs are a distinct population of brain resident macrophages located within CNS interfaces, which include the perivascular spaces (perivascular macrophages, PVMs), the leptomeninges (meningeal macrophages, MMs), and the choroid plexus (choroid plexus macrophages, ChPM) and are identified in human and mouse brain as CD163^+^ and CD206^+^ cells, respectively [12]. Recent studies have indicated that CAMs are involved in a wide variety of pathological states [8, 9, 23, 48]. Their main functions, as described to date, include regulation of blood–brain barrier (BBB) integrity and cerebrospinal fluid (CSF) dynamics, phagocytosis of blood-borne pathogens, and control of leukocyte migration [10, 27]. In addition, predominant cells found in the human IA walls are CD163^+^, suggesting their significant role in wall degeneration [16]. Our recent work has confirmed the accumulation of CD206^+^ cells in a murine model of IAs [31]. Furthermore, in a previously published mouse model of hypertension, PVMs were found to produce large amounts of reactive oxygen species (ROS), contributing to hypertension-induced neurovascular disorders and BBB leakage [13, 43], while PVM depletion restored cognitive functions. As a dominant source of proinflammatory cytokines and chemokines, PVMs have also been reported to mediate neutrophil recruitment following bacterial infection [1].

Here, we report a novel mouse model of IAs at the MCA that exhibits pathological characteristics similar to human aneurysms, including morphological and inflammatory changes. Our data demonstrate that vascular inflammation within the IA area, as assessed by molecular MRI, precedes and predicts aneurysm rupture, highlighting this technique as a promising predictive and diagnostic clinical tool in patient follow-up studies.

Additionally, we reveal that CAMs play a crucial, inflammatory role in IA formation and rupture. Indeed, CAM depletion by clodronate (CLO) liposomes reduces the formation of IAs, IA rupture rate, hemorrhage volumes and the number of neutrophils, in our murine model of MCA aneurysms. Our data suggest a previously unexplored role of CAMs as central actors orchestrating inflammation in the IA walls and promoting IA ruptures. Thus, we propose vascular inflammation as a potential diagnostic marker for the clinical management of unruptured IAs, and CAMs as important targets for novel therapeutic strategies in the treatment of aneurysms prone to rupture.

## Results

### A new mouse model of MCA IAs

**Fig. 1:**
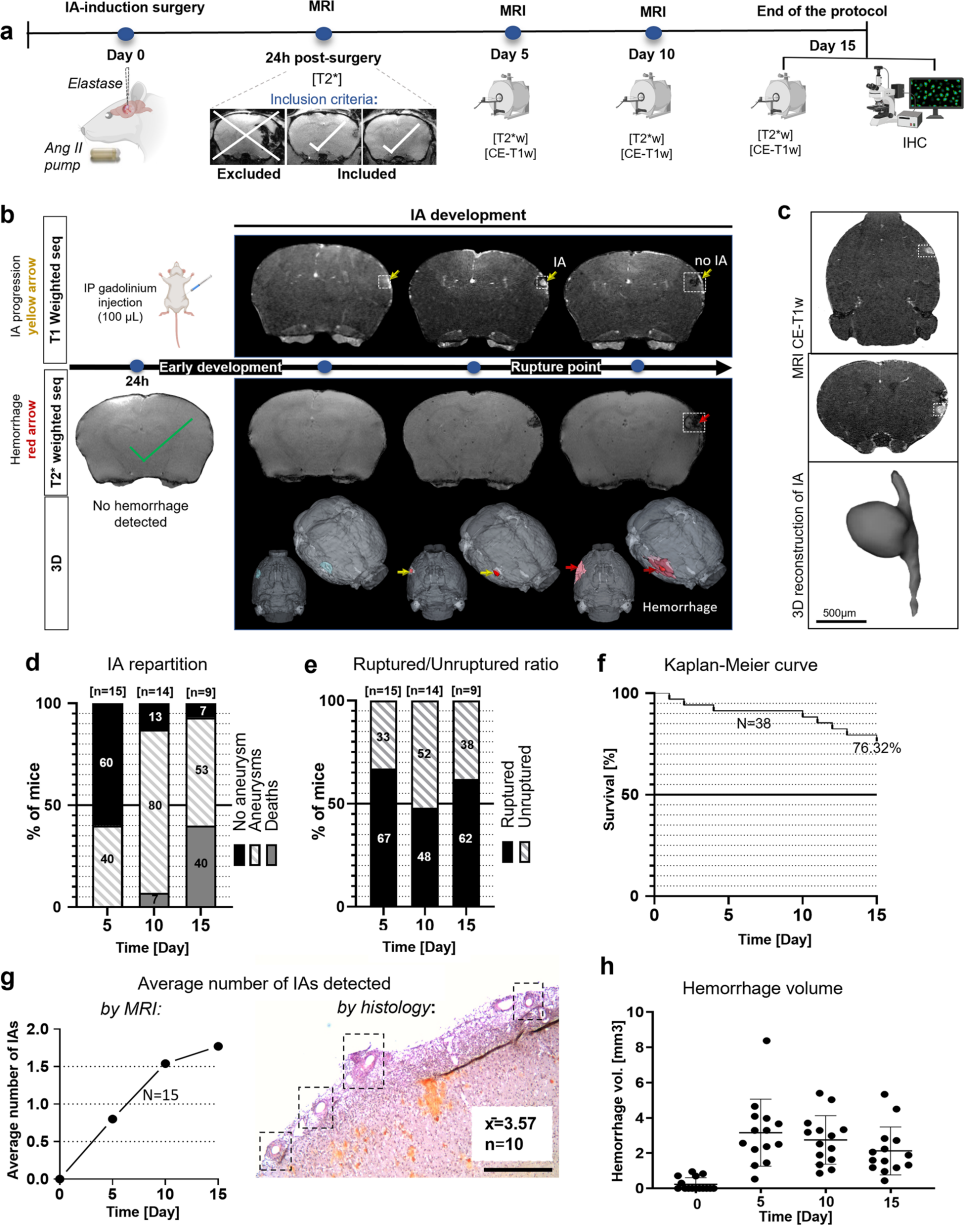
A new mouse model of IAs at the middle cerebral artery. **a** Schematic representation of experimental design and the timeline scale. **b** Representative images of formation, growth and rupture assessed by longitudinal MRI. Contrast-enhanced T1 weighted (CE-T1-w) MRI was used to confirm the presence of IAs (yellow arrow). T2*-w MRI was performed to confirm the rupture of IAs with the presence of hemorrhage (red arrow). Once IA ruptures, it is no longer observable with T1-w MRI, bleeding appears as hypointense signal on T2*-w MRI. **c** Representative T1-w images, with the location of the IA at the lateral part of the brain. Three-dimensional IA reconstruction created from the MR scans revealed the saccular type of IAs. **d** Occurrences of ruptured and unruptured aneurysms at different time points revealed that many aneurysms appear already at the Day 5, and most of them are ruptured by the Day 15 (presented in **e**). **f** Kaplan–Meier survival graph. **g** Average number of IA per animal quantified from T1-w MRI during the 15-days timeline (left), average number of IAs per animal assessed per histological analysis at the end of the protocol (right). Scale bar: 500 µm. **h** Hemorrhage volumes measured from T2*-w scans during the experimental timeline

The formation of IAs was induced by local microinjection of elastase behind the MCA bifurcation (Fig. 1a; Additional file 1: Figure 1a) followed by subcutaneous Angiotensin II (Ang II) osmotic mini-pump insertion (Fig. 1a; Additional file 1: Figure 1a). Local microinjection of elastase induced vascular wall degradation while subcutaneous infusion of Ang II increased blood pressure from day 3, reaching a plateau by day 10 post pump implantation (Additional file 1: Figure 1b, c). The dose of elastase was chosen after a dose–response experiment (Additional file 1: Figure 2a). During our model development phase, we performed experiments without pump insertion and still observed the presence of aneurysms, however with smaller formation and rupture rate (Additional file 1: Figure 2b–d).

The development of IAs in mice was determined by contrast-enhanced T1-weighted (CE-T1-w) MRI following the injection of gadolinium (DOTAREM) as a contrast agent at three different time points (Day 5, Day 10, and Day 15; Fig. 1a, b; Additional file 1: Figure 1e, f). IAs were counted by two different experimenters observing the intravascular accumulation of gadolinium within the MCA using the CE-T1w MR scans (Fig. 1b; Additional file 1: Figure 1g). The 3D reconstructions of IAs from the MR scans revealed saccular subtype aneurysms (Fig. 1c). The spontaneous rupture of the IAs resulted in either subarachnoid or intracerebral bleeding (Additional file 1: Figure 1d). Forty percent of IAs were already present at the Day 5 (Fig. 1d), and 62% were ruptured by Day 15 (Fig. 1e). At the end of the protocol, 93% of mice developed IAs (Fig. 1d), and the overall survival was 76% (Fig. 1f). The average number of IAs per mouse was estimated to be 2 and 3.57 as observed through MRI and histological analysis, respectively (Fig. 1g). Mice were mainly asymptomatic even after rupture, probably due to the specific location of the aneurysms, i.e., the outer cortical area, and the relatively small hemorrhage volumes in the case of rupture (Fig. 1h).

**Fig. 2:**
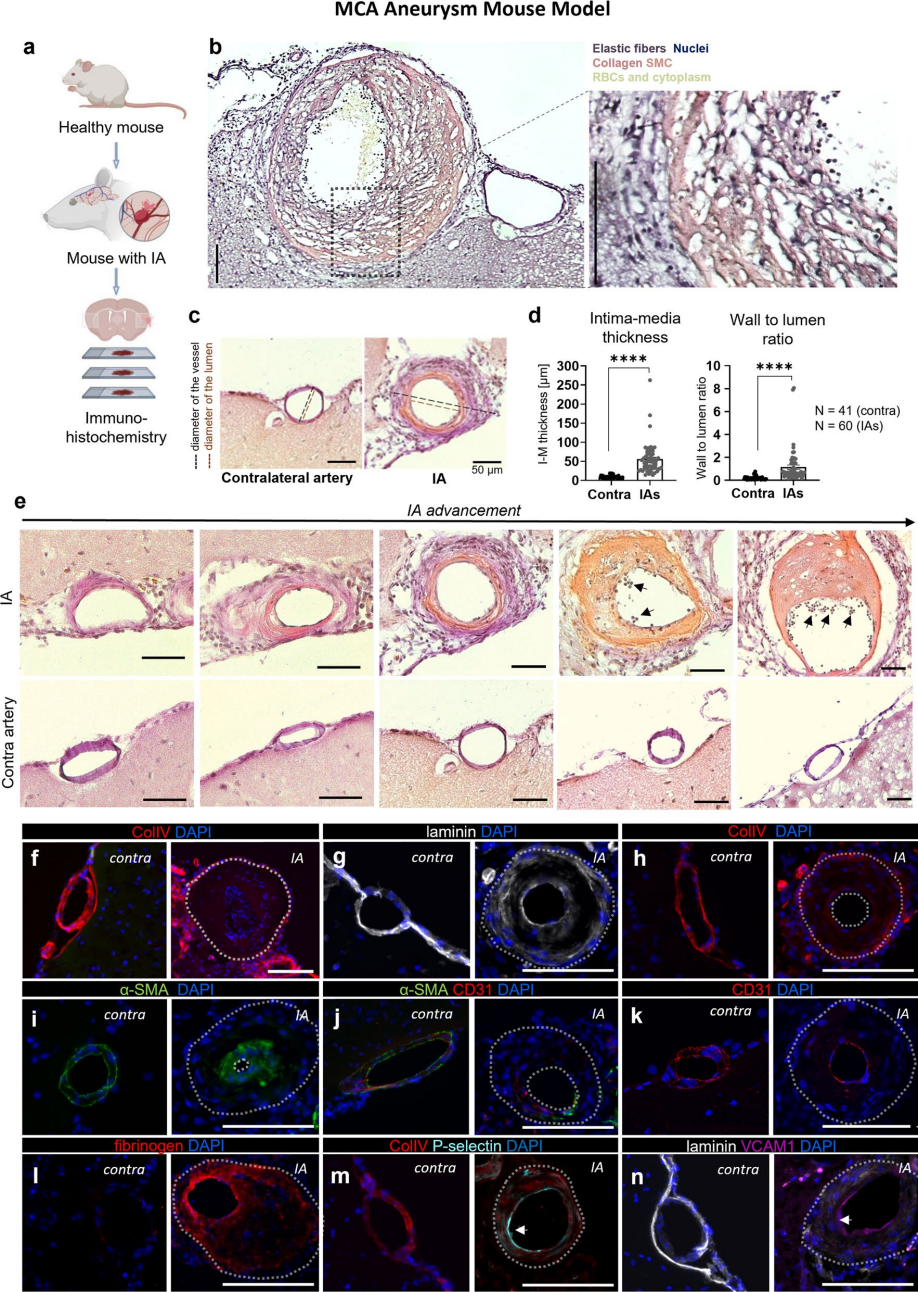
Histological and immunofluorescence analysis of vessel morphological changes in the mouse IA samples. **a** Schematic representation of experimental design. **b** Modified Verhoeff-Van Gieson staining on cryosections from the mouse brain, continuously infused with Ang II for 14 days. Samples were obtained at Day 15. Elastic laminae are visualized by the dark purple lines in the media of the artery. Collagen appears red/purple, nuclei in black and other structures in yellow. IA vessel shows visible degradation of internal elastic lamina, collagen turnover in red, and cell accumulation (nuclei in black). Closer look at the aneurysm (20× magnification, on the right). Representative section of IA in greater detail revealed multi-lobed nucleus characteristic for neutrophils—found at the lumen of the IA vessel. Scale bar: 100 µm. **c** Representation of how the measurements were carried out (Scale bar: 50 μm). **d** Intima media thickness and wall-to-lumen ratio. Based on the histological images, the widest vessel and vessel lumen diameters of both IAs and the contra arteries were measured. Vessel wall diameter was defined as the difference in intima-media thickness. Both intima-media thickness and the wall-to-lumen ratio were significantly increased in the aneurysms compared to the contra arteries. (****P < 0.0001, N = 11 mice, n = 60 aneurysms/n = 41 contra arteries, Mann–Whitney U test). **e** Representative images of Verhoeff–Van Gieson staining with IAs assigned different stages of the progression according to the manifested morphological changes. Inflammatory cell infiltration in the IA walls and at the luminal surface are indicated with the arrow. Scale bar: 50 µm **f** Vessel wall thickness—aneurysm vessel wall shows substantially increased diameter in compared to the healthy, contralateral artery. **g** Weak and dispersed laminin signal in the IAs as a sign of internal laminae degradation. Visible difference in laminin expression between the healthy contralateral artery and the IA. **h** Confirmation of Col IV turnover (characteristic for IAs) with Col IV positive signal in the aneurysm vessel. **i** SMC proliferation and migration. **j** Some IAs presented a complete degradation of SMCs. **k** Damage of the endothelium characteristic for IAs. CD31 (platelet endothelial cell adhesion molecule; PECAM-1) marker reveals discontinuous signal in the IA vessel wall with no cell–cell junctions were clearly visible. **l** Fibrinogen accumulation on the luminal side of the IA. **m, n** Immunofluorescence images with visible P-selectin signal (in turquoise blue, **m**) and VCAM-1 (magenta pink, **n**) signal, characteristic of increased endothelial activation found within the vessel lumen**.** Scale bar: 100 µm. Statistical analysis was performed using Mann–Whitney U test; **P < 0.01, ***P < 0.001; NS, not significant. Further detailed information on statistics is provided in the Methods and Materials

Histological analysis of the brain sections was performed using the elastica van Gieson staining to visualize connective tissue and elastic fibers (Fig. 2a, b). Analysis of the vessel wall (intima-media) thickness and wall-to-lumen ratio showed significant increases in both parameters in the IAs compared to the contralateral arteries (Fig. 2c, d), confirming the data observed by MRI. Furthermore, the internal elastic lamina (IEL) was mostly damaged, fragmented, or completely degraded in our samples of induced mouse IAs (Fig. 2b). The collagen layer appeared thickened as compared to the control vessels and was most accentuated in the advanced IAs in mice (Fig. 2b, e). A large number of infiltrating cells was observed in all of our IA samples, with their numbers increasing in line with the severity of IA advancement (Fig. 2e).

**Fig. 3:**
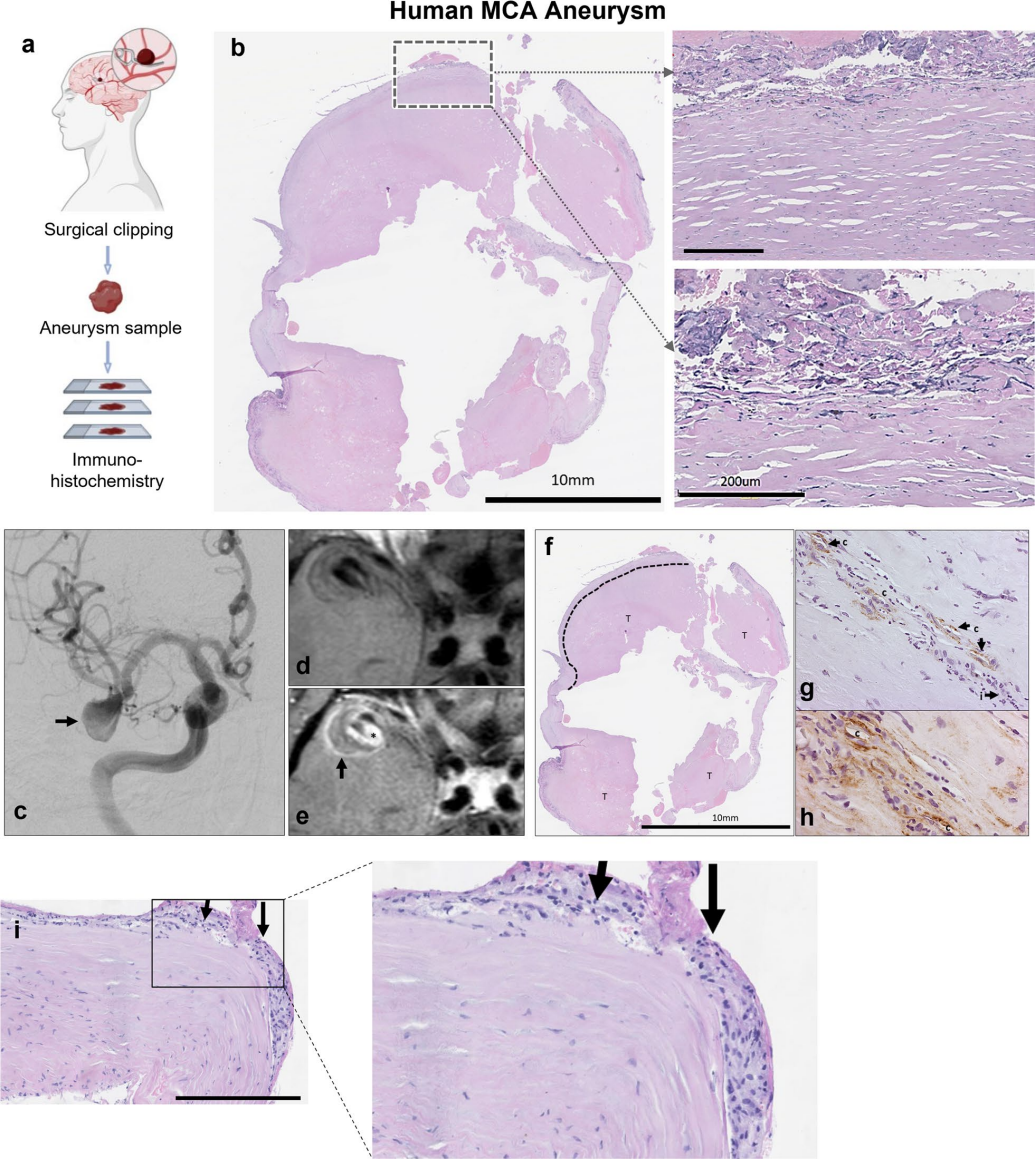
Histological analysis of vessel morphological changes in the human IA samples. **a** Schematic representation of experimental design. A human sample was resected after microsurgical clipping from a partially thrombosed aneurysm arising from the bifurcation of the right middle cerebral artery. **b** Hematoxylin eosin staining of human IA sample obtained after clipping reveals thick, hypocellular wall structure with thrombus on the luminal surface. Increased thickness of the vessel wall and the wall decellularization (loss of mural cells) confirms the findings from the mice MCA aneurysms. Scale bar: 10 mm and 200µ. **c** Coronal view of the preoperative digital subtraction angiography (on the left) shows the filling lumen of the IA (marked with an arrow), while preoperative T1-w black blood MRI sequence without (in **d**) and with gadolinium contrast (in **e**). Wall enhancement observed after gadolinium enhancement is marked with arrow in **e**. **f** Hematoxylin eosin staining (0.7× magnification) demonstrates that the thickness of IA wall is largely composed of thrombus lining the luminal surface (marked with T). The border between the actual wall and the thrombus is depicted with an interrupted line. Higher magnification microphotographs from a CD31 immunofluorescence in **g** and from a CD34 immunofluorescence in (**h**). **g**, **h** with 20× magnification and 40× magnification, respectively. The presence of inflammatory cell infiltration is marked with **i** and capillaries are marked with **c** (both presented in **g**). **i** Adventitial inflammatory cell infiltration within the wall. The dark rectangle in **b** corresponds to the wall region from with the higher magnification microphotographs in panel **i** are taken. Similarly, the rectangle in **i** correspond to the wall regions from which the higher magnification microphotographs were taken. Scale bars: 500 µm, and 200 µm respectively

MR scans were used as anatomical reference to detect the IAs in the histological and immunofluorescent (IF) brain sections (Additional file 1: Figure 2e). IF analyses confirmed the prominent IA features, most noticeably increased vessel wall thickness (Fig. 2f) and a low signal of both laminin and collagen type IV (Col IV) (Fig. 2g, h). α-SMA, a marker of smooth muscles cells (SMCs), was mostly increased in the IA walls due to the proliferation of SMCs (Fig. 2i). However, in some samples, almost complete degradation of SMCs was found, suggesting progressed vessel wall degeneration (Fig. 2j). All aneurysms showed a damaged endothelial cell (EC) layer as manifested by a discontinuous CD31^+^ signal where no cell–cell junctions were clearly visible (Fig. 2k). The presence of a fibrinogen signal in the vessel wall of some of the IA samples suggested BBB disruption (Fig. 2l). Both P-selectin^+^ and VCAM-1^+^ signals were found at the luminal EC side in the IAs whereas they were mostly absent in the contralateral arteries (Fig. 2m, n). The IAs formed at the elastase injection site in mice (Figs. 1a–c, 2b, e) had a pathophysiological appearance similar to that observed in the humans through radiology and histology (Fig. 3a–i), in terms of increased thickness of the vessel wall, inflammatory cell infiltration, and vessel wall decellularization (Fig. 3b, f–i).

### Vascular inflammation, non-invasively detected by molecular MRI, is predictive of bleeding severity after IA rupture

**Fig. 4:**
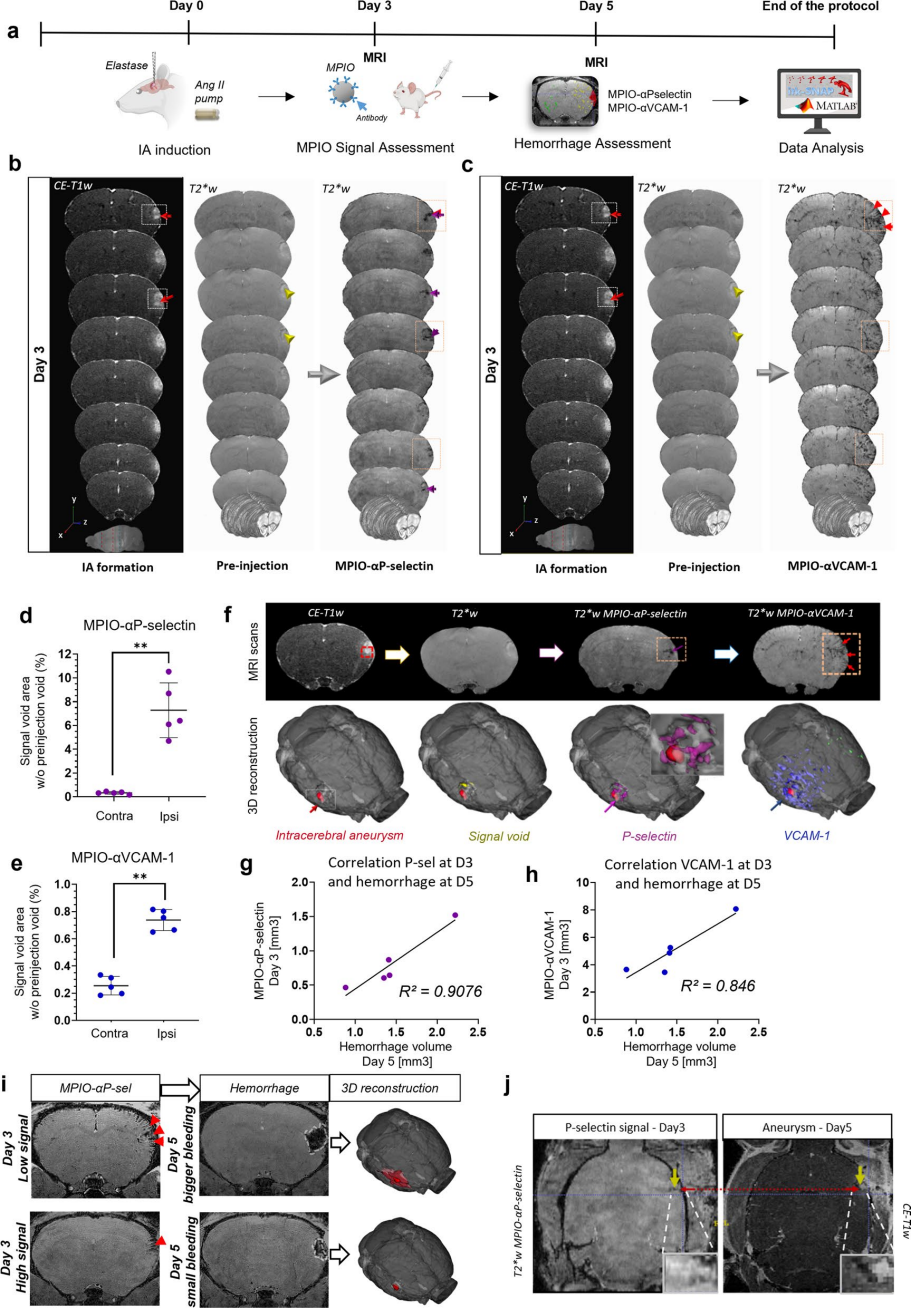
Molecular MRI reveals vascular inflammation in the IA area and predicts the outcome severity. **a** Schematic representation of the experimental approach. **b** Representative high-resolution T2*-w images (coronal view) before and after MPIO-αP-selectin intravenous injection in mice at Day 3 post-IA induction. MPIO-αP-selectin–induced hypointense signal in the T2*-w sequence in the vicinity of the IA area. Arrowheads indicate the MPIO-specific hypointensities. P-selectin endothelial activation is increased in the ipsilateral hemisphere and restricted to the IA area. (**P < 0.01; N = 5 mice/group; Mann–Whitney U test). **c** Representative high-resolution T2*-w images (coronal view) after MPIO-αVCAM-1 intravenous injection in the mice subjected to IA induction, at Day3. Arrowheads indicate the MPIO-specific hypointensities concentrated mainly around the IA area. **d** Corresponding signal void quantification for the MPIO-αP-selectin (**P < 0.01; N = 5 mice/group; Mann–Whitney U test). **e** Corresponding signal void quantification for the MPIO-αVCAM-1. VCAM-1 endothelial activation significantly increased in the ipsilateral hemisphere and restricted to the IA area (**P < 0.01; N = 5 mice/per group; Mann–Whitney U test). **f** Representative three-dimensional reconstruction of MR scans (T2*-w sequence) with MPIO signal voids and their distribution across the brain. **g** Correlation between the P-selectin signal from the MR scans at Day3 and hemorrhage volumes at Day5 (N = 5, R^2^ = 0.91). **h** Correlation between the VCAM-1 signal from the MR scans at Day3 and hemorrhage volumes at Day5 (N = 5, R^2^ = 0.85). **i** Representative images of P-selectin MPIO signal void at earlier time points (Day 3) and the hemorrhage volumes after the IA ruptures (Day 5). A smaller volume of signal void results in a small hemorrhage, whereas a larger volume results in a substantially bigger hemorrhage. **j** Colocalization of IA location at Day5 and the P-selectin signal from the MR scans at the Day3. Statistical analysis was performed using Mann–Whitney U test; **P < 0.01, ***P < 0.001; NS, not significant. Further detailed information on statistics is provided in the Methods & Materials

To determine a potential link between endothelial cell activation and IA rupture, we performed in vivo longitudinal molecular MRI after the injection of microparticles of iron oxide (MPIOs) coupled to P-Selectin or VCAM-1 antibodies at different time points during pathology progression (Fig. 4a; Additional file 1: Figures 3, 4). No P-Selectin or VCAM-1 MPIO signal voids were observed prior to the MPIO injection (Fig. 4b, c) at any of the time points. MPIOs were progressively cleared from the brain, resulting in negligible background 24h after each MPIO-injection, allowing longitudinal studies by repetitive assessment of endothelial activation. Three days after IA induction, positive signal voids were observed specifically at the IA area for both MPIO-αP-selectin (Fig. 4b, d, f) and MPIO-αVCAM-1(Fig. 4c, e, f), with latter also partly detected at a relative distance from the IA site (Fig. 4c, e, f). Interestingly, MPIO signal voids found at Day 3 correlated with the hemorrhage volumes after IA ruptures observed at Day 5 (Fig. 4g–i), with an *R*^*2*^ value of 0.91 for MPIO-αP-selectin (Fig. 4g) and 0.85 for MPIO-αVCAM-1 (Fig. 4h). Furthermore, the MPIO-αP-selectin signal present at earlier time point (Day 3) was situated at the site of IA formation at the subsequent time point (Day 5; Fig. 4j).

### CAMs accumulate in the IA wall in both humans and mice

**Fig. 5:**
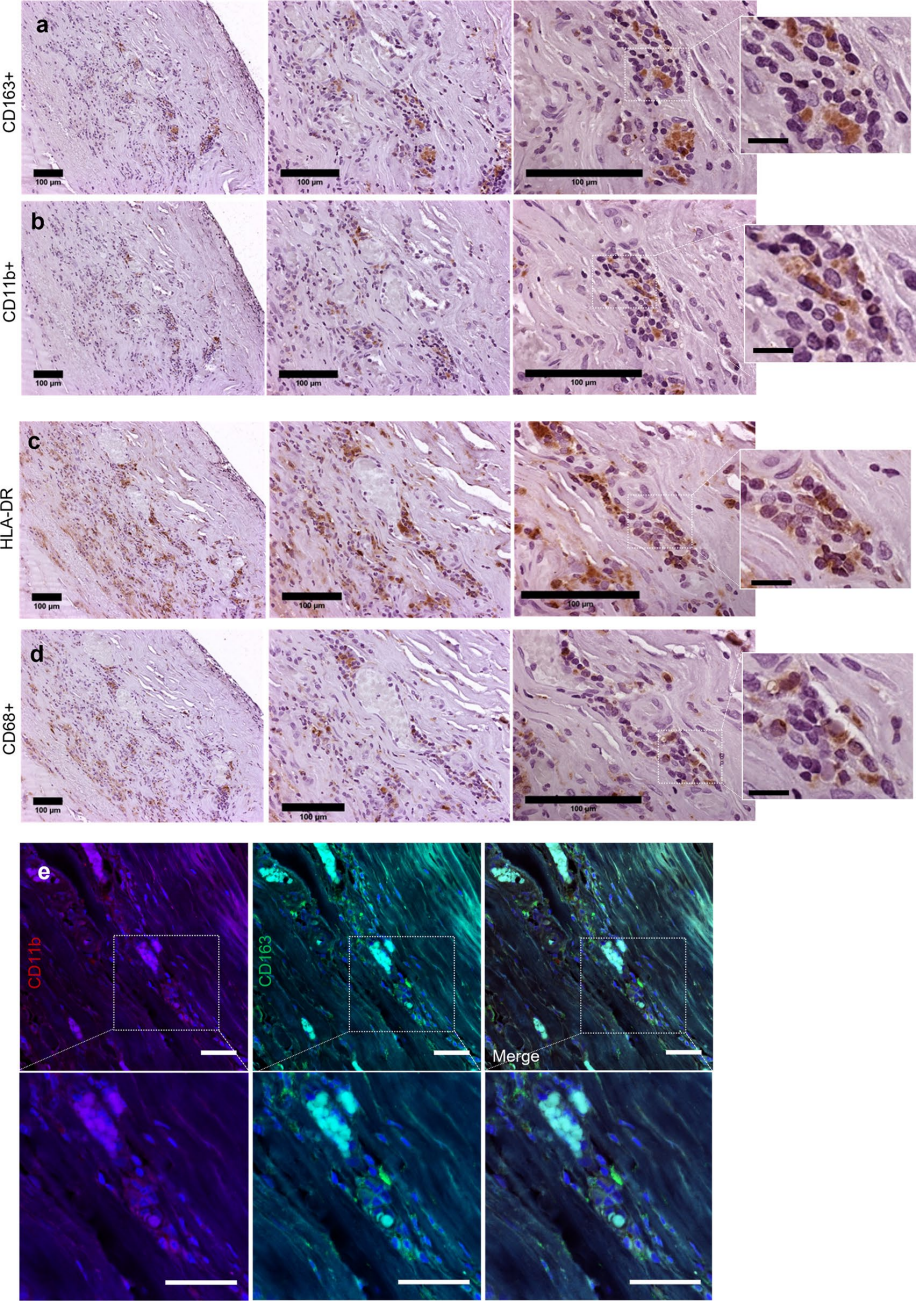
CD163^+^ Cd11b^+^ macrophage accumulation in the human IA wall. **a**, **b** Histological analysis of human MCA aneurysm wall reveals cells expressing the scavenger receptor CD163 as well as the CD11b^+^ cell presence in the IA wall. These represent the most abundant inflammatory cell types in our human IA sample. **c** A population of HLA-DR^+^ increased within the wall whereas some of these cells were also CD68 positive (as shown in **d**). **e** CD163 and Cd11b double-staining reveals colocalization of these cells in the human IA wall

To assess the similarity in pathophysiological appearance between IAs in our mouse model and our human samples (Figs. 2, 3), we verified whether the number of CAMs found nearby and within the IA wall was similar both in human and mouse samples by using classical histological markers for CAMs such as CD163 (in human samples) or CD206 (in mouse samples), CD11b, CD68 and HLA. The human IA samples showed an accumulation of CD163^+^ and CD11b^+^ cells (Fig. 5a, b), as well as presence of HLA-DR^+^ myeloid cells (Fig. 5c) and CD68+ cells (Fig. 5d) within the IA wall. In addition, some of the CD163^+^ cells colocalized with CD11b^+^ (Fig. 5e).

**Fig. 6:**
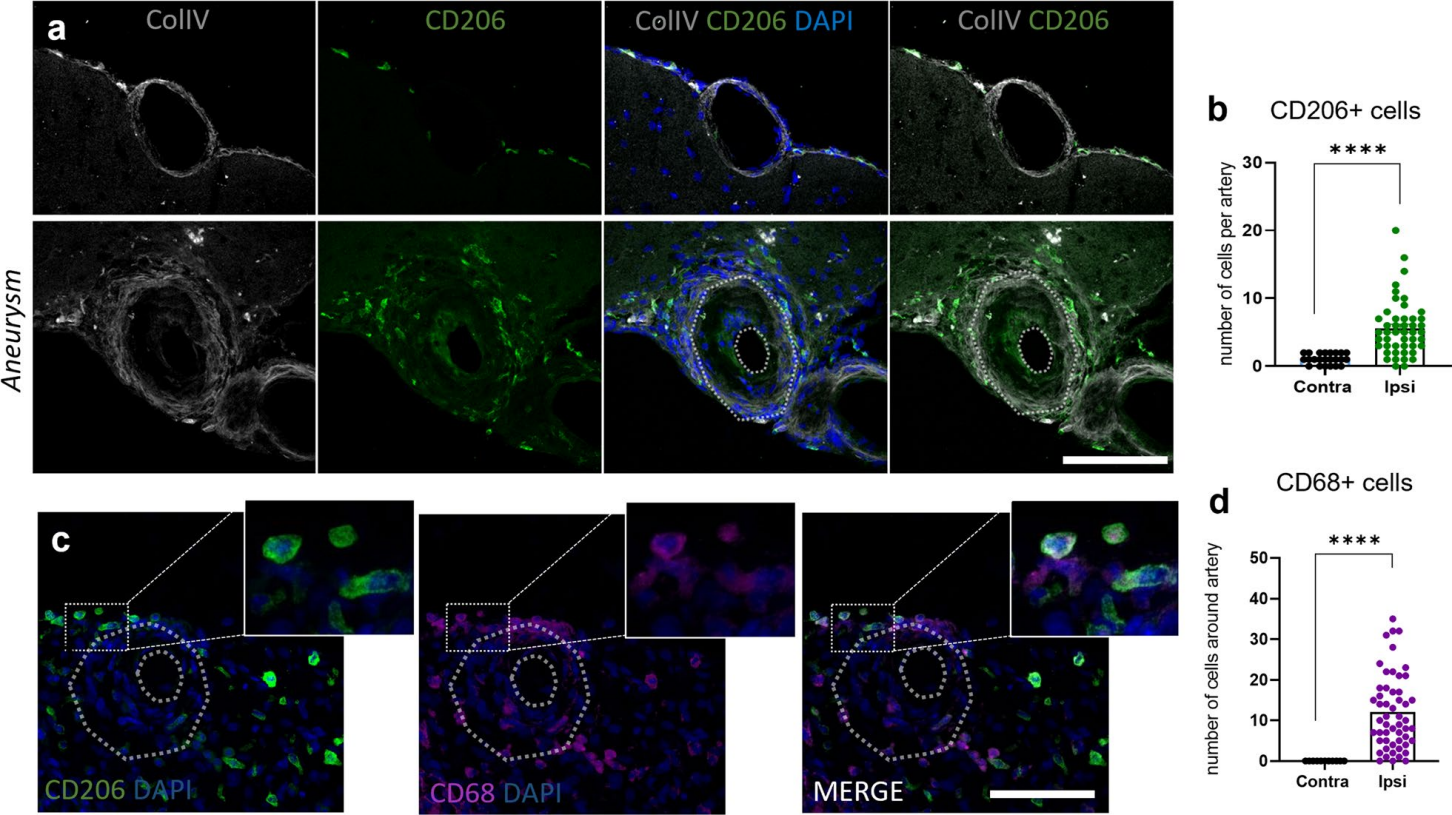
CD206^+^ CAM accumulation in the mouse IA wall. **a** An increased number of CD206^+^ macrophages found in the ipsilateral hemisphere at Day5 post-aneurysm induction, especially around the IAs. This was significantly higher than the number of CD206^+^ cells found around the control vessels. **b** Quantification of accumulated CAMs confirmed the significant accumulation of these cells in the IA area (****P < 0.0001, N = 11 mice, n = 47 IAs/n = 23 contra arteries, Mann–Whitney U test). **c** CAMs (CD206^+^ cells) are positive for the lysosomal marker CD68. Scale bar: 100 μm. Scale bar: 100 μm. **d** Quantification of CD68^+^ cells in the IA area. (****P < 0.0001, N = 11 mice, n = 47 IAs/n = 23 contra arteries, Mann–Whitney U test). Statistical analysis was performed using Mann–Whitney U test; **P < 0.01, ***P < 0.001; NS, not significant. Further detailed information on statistics is provided in the Methods and Materials

Similar results were observed in the mouse IA samples, where a high accumulation of CD206^+^ cells was observed nearby and in the IA walls as early as day 5 of the IA development stage (Fig. 6a, b). In the contralateral hemisphere, CD206^+^ cells were found exclusively in the perivascular spaces (corresponding to PVMs) and the meninges (corresponding to MMs) (Fig. 6a). In contrast, in the IA area, CD206+ cells were highly accumulated (Fig. 6b) with a subset showing a weak positivity for the lysosomal marker CD68 (Fig. 6c, d). Taken together, these data indicate that CD163^+^ (humans)/CD206^+^ (mouse) macrophages proliferate and/or infiltrate the IA site in both humans and mice.

**Fig. 7:**
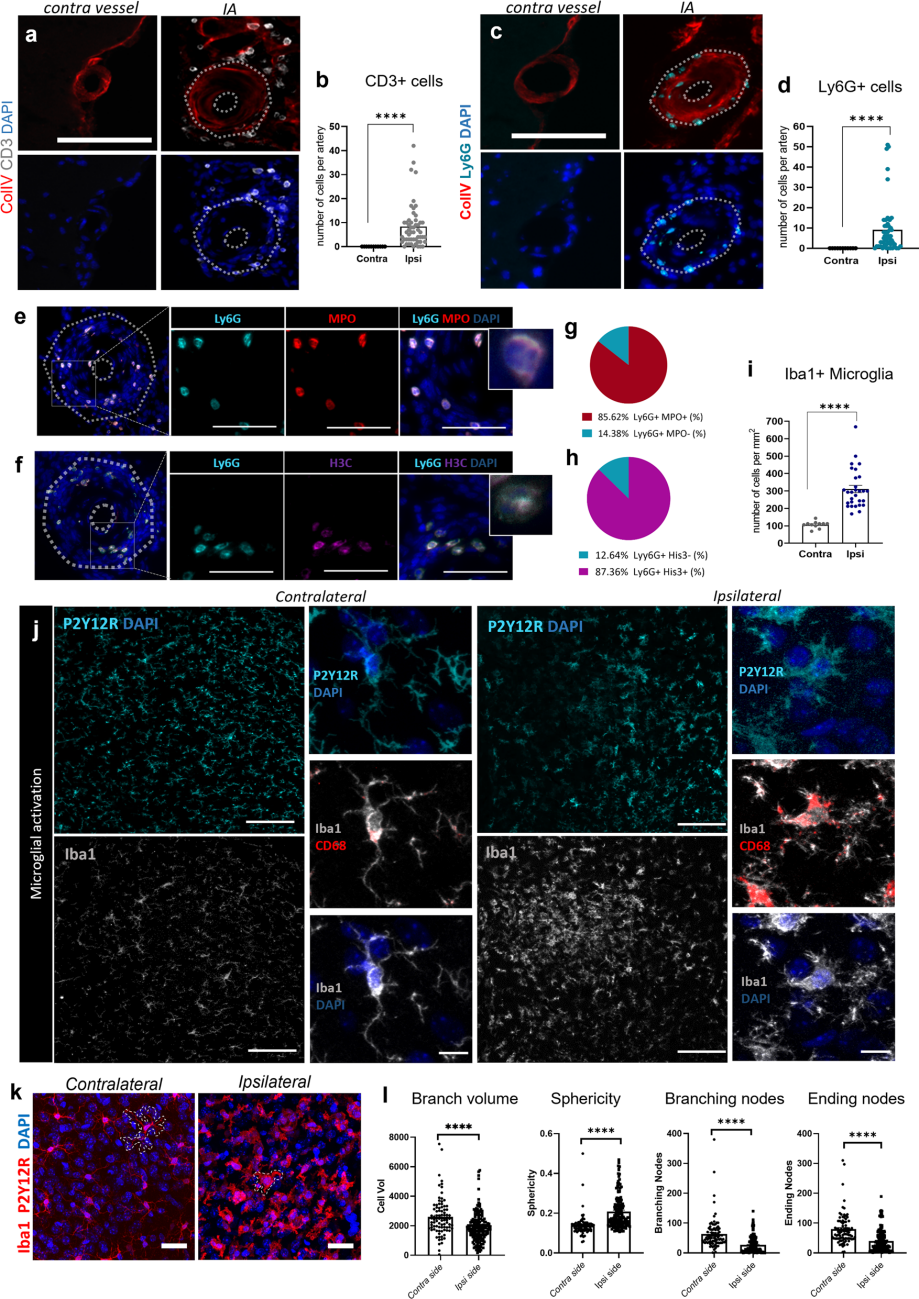
Inflammatory cell accumulation in the mouse IA wall at Day 15 post-aneurysm induction. **a** Representative image of CD3^+^ cell accumulation around the IA vessel. Scale bar: 100 μm. **b** Quantification confirmed increased infiltration of CD3^+^ T-cells in the IA area. N = 11 mice, n = 53 IAs/n = 11 contra arteries. ****P < 0.0001, Mann–Whitney U test. **c** Representative image of Ly6G^+^ cells infiltrating the IA vessel wall. Scale bar: 100 μm. **d** Quantification confirmed increased infiltration of Ly6G^+^ neutrophils in the IA area. N = 11 mice; n = 51 IAs/n = 11 contra arteries. ****P < 0.0001, Mann–Whitney U test. **e, f** Infiltrating neutrophils co-localize with MPO and H3Cit positive signal. Scale bar: 50 μm. **g** Proportion of Ly6G^+^ MPO^+^ cells colocalization in the human MCA IA sample. **h** Proportion of Ly6G^+^ H3Cit^+^ cells in the human MCA IA sample. **i** Iba^+^ cell quantification shows increase in the number of microglia around the IA area. ****P < 0.0001. N = 6 mice, n = 29 IAs/n = 11 contra arteries, Mann–Whitney U test. **j** Microglial activation in the IA area confirmed with the expression of specific microglial activation markers. Scale bar: 100 μm and 10 μm, respectively. CD68 lysosomal marker was used to confirm the active microglia. Unlike the resting form of microglia found around the arteries on the contralateral hemisphere with long branched processes, microglia near the IA site show round shape, with decreased process extension. Closer to the IA, the expression of P2Y12R is found to be decreased, while expression of Iba1 marker is still highly present. **k, l** Unbiased automatic analysis of microglia morphological confirms the activation in the area of IAs. A significant decrease in the branch volume, branching and ending nodes and the increase in the sphericity was found ipsilaterally, in compared to the contralateral side. (****P < 0.0001, N = 5 mice/group. Kruskal–Wallis test followed by Dunn’s multiple comparisons test). Further detailed information on statistics is provided in the Methods and Materials

We also identified other blood-borne immune cell subtypes in the IA site at a late stage of the IA formation (15 days post IA induction surgery), including CD3^+^ T-cells (Fig. 7a, b) and Ly6G^+^ cells (neutrophils; Fig. 7c, d). Ly6G+ cells expressed myeloperoxidase (MPO) and citrullinated histone H3 (H3Cit), demonstrating the presence of neutrophil extracellular traps (NETs; Fig. 7e, f). Indeed, 85.62% and 87.36% of Ly6G^+^ cells were positive for MPO and H3Cit, respectively (Fig. 7g, h).

Microglial activity changes were determined both morphologically and phenotypically. Quantitative analysis revealed a significantly higher number of Iba1^+^/P2Y12R^+^ cells at the ipsilateral site compared to the contralateral side (Fig. 7i, j). Phenotypically, the expression of P2Y12R was decreased in areas close to the IA, whereas the expression of Iba1 was increased (Fig. 7j). CD68, a lysosomal marker associated with microglial phagocytic activity, was also highly expressed in the microglial cells in the ipsilateral hemisphere (Fig. 7j). Microglial response to IA was also confirmed with an unbiased automated analysis of microglial morphology, which revealed increased sphericity of the soma and a decrease in the number of processes, and filopodia at the IA site, as opposed to microglia assessed around the corresponding arteries in the contralateral hemisphere. (Fig. 7k, l).

### CAMs participate in IA formation, rupture rate and neutrophil homing in the IA wall

**Fig. 8:**
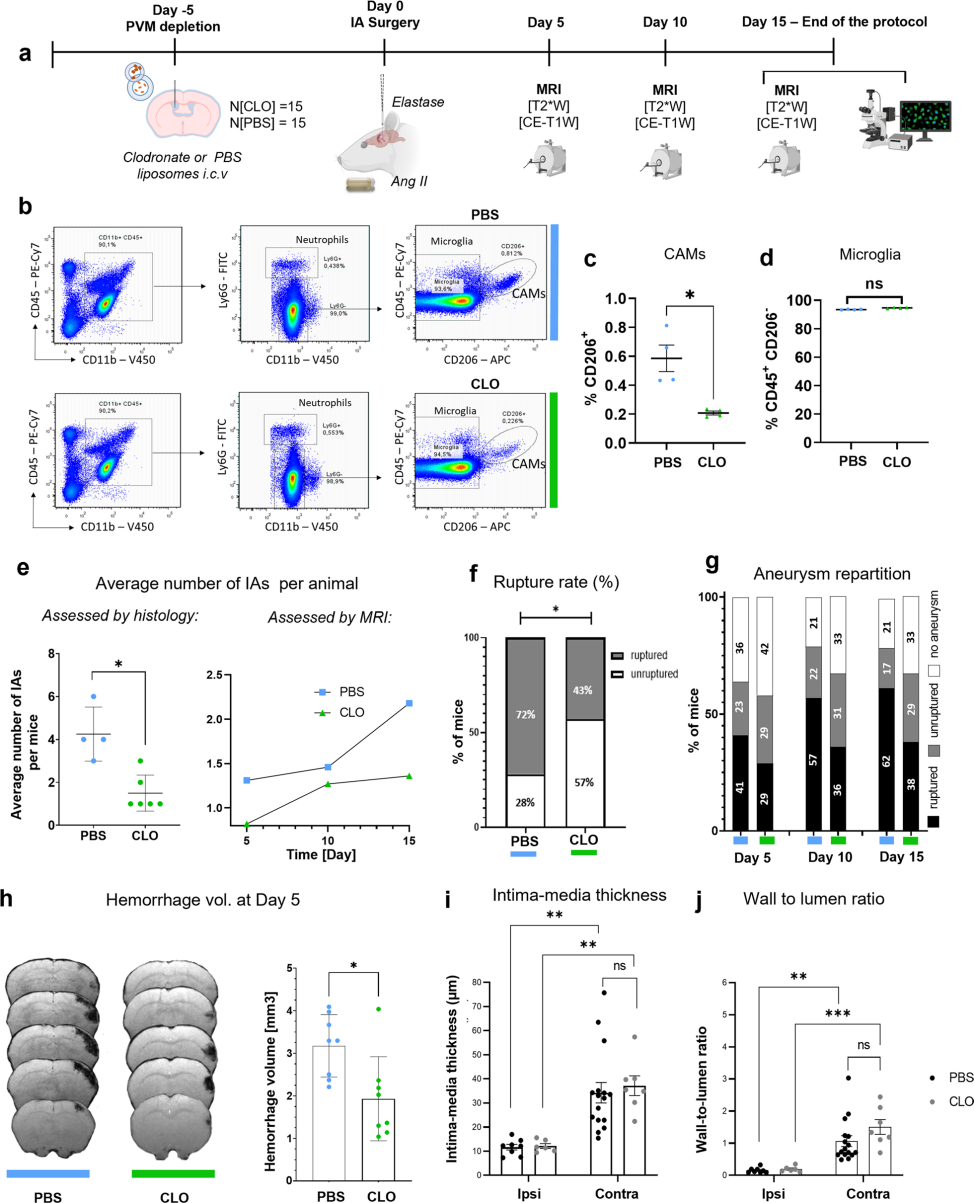
CAM depletion reduces IA formation and ameliorates severity of IA ruptures. **a** Schematic representation of the experimental design. **b** Representative flow cytometry dot-plots and gating strategy used for quantification of CD206^+^ macrophages and microglia, with vehicle or clodronate-liposomes. **c** CLO-liposomes significantly reduce the number of CD206^+^ macrophages even 10 days post-depletion, whereas microglia number stayed unaltered. Flow cytometry quantification of CD206^+^ macrophages and Iba1^+^ microglia (**d**), treated either with vehicle or CLO-liposomes (N = 4, *P < 0.05, Mann–Whitney U test). **e** Average number of IAs developed per animal. Immunofluorescence analysis data (on the left) show that CLO-treated mice develop significantly fewer IAs compared to the control group (*P < 0.05, N = 6 CLO-treated mice/N = 4 PBS-treated mice, Mann–Whitney U test). Longitudinal MR studies over the timeline of 15 days confirmed the existing difference (on the right). **f** CLO-treated mice have decreased global rupture rate (*P < 0.05, n = 14 PBS-treated mice/n = 13 CLO-treated mice; PBS versus CLO: 72% versus 43%, Fisher exact test). **g** Aneurysm repartition between the PBS and CLO-treated mice during the period of 15 days (assessed on MR scans). **h** CAM depletion significantly reduces the hemorrhage volumes after the IA rupture, assessed by the MRI scans from T2*-w sequences (*P < 0.01; Mann–Whitney U test). **i**, **j** Wall-to-lumen ratio and intima-media thickness comparison between the group revealed no significant difference in dimensions of IAs (**P < 0.01, ***P < 0.001, N = 4 CLO-treated mice, n = 7 IAs/n = 6 contra arteries; N = 4 PBS-treated mice, n = 16 IAs/n = 8 contra arteries. Two-way ANOVA). Statistical analysis was performed using two-way analysis of variance (ANOVA) with post hoc Dunnett adjustment; *P < 0.05, **P < 0.01, ***P < 0.001. Further detailed information on statistics is provided in the Methods & Materials

Given the finding of CAM accumulation in the IA vessel walls (Fig. 6), we depleted CAMs by injecting clodronate (CLO) liposomes in the lateral ventricle 5 days before IA induction surgery to elucidate their role in IA pathophysiology (Fig. 8a). We observed a specific depletion of CAMs (CD11b^+^ CD45^+^ CD206^+^ cells) 10 days after the CLO injection (Fig. 8b, c), which did not affect microglia numbers (CD11b^+^ CD45low; Fig. 8d). As previously reported [11], clodronate liposomes reduced at least 70% of the total number of CAMs compared to the vehicle (Fig. 8b).

**Fig. 9:**
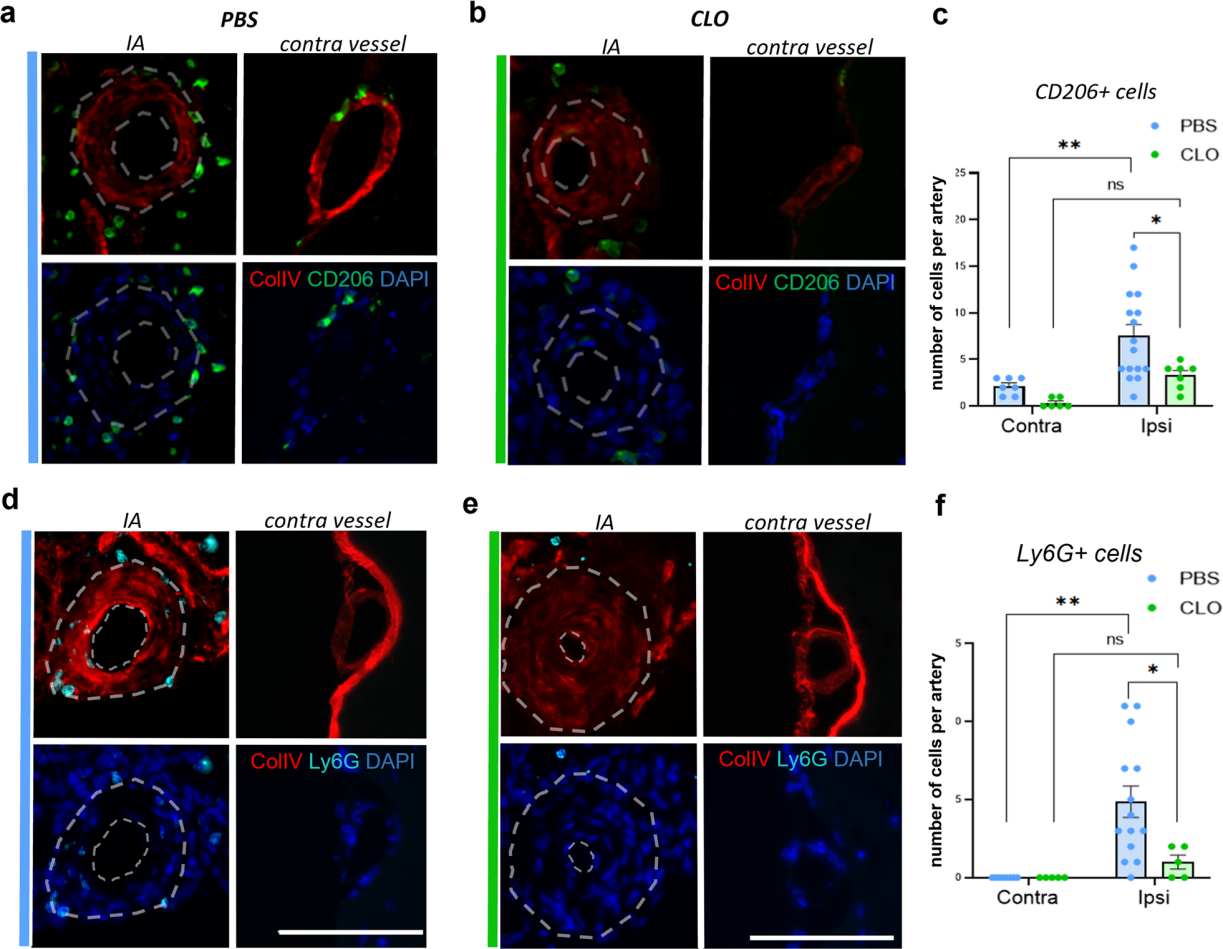
CD206^+^ and Ly6G^+^ cell quantification in IA walls of PBS and CLO-treated mice. **a** Representative image of CAM accumulation in PBS-treated mice. **b** Representative image of CAM absence in CLO-treated mice. **c** Quantification of CAM number around the IA vessel wall and the contra arteries at Day5 post-aneurysm induction (N = 4 CLO-treated mice, n = 7 IAs/n = 6 contra arteries. N = 4 PBS-treated mice, n = 16 IAs/n = 7 contra arteries). **d** Representative image of neutrophil accumulation in PBS-treated mice. **e** Representative image of the reduced neutrophil number in CLO-treated mice. **f** Quantification of neutrophil number in the IA vessel wall at Day5 post-aneurysm induction (N = 4 CLO-treated mice, n = 5 IAs/n = 5 contra arteries. N = 4 PBS-treated mice, n = 14 IAs/n = 8 contra arteries. *P < 0.05, **P < 0.01, two-way ANOVA. Scale bar: 100 μm). Statistical analysis was performed using two-way analysis of variance (ANOVA) with post hoc Dunnett adjustment. Further detailed information on statistics is provided in the Methods and Materials

Longitudinal MRI was conducted on both PBS- (control) and CLO-injected mice to assess IA formation and hemorrhage volumes at day 5, day 10, and day 15 post-surgery (Fig. 8a). CAM-depleted mice developed fewer IAs compared to the control group, as indicated by both MRI and immunofluorescence analyses (Fig. 8e). Additionally, CAM-depleted mice had a significantly lower rupture rate (Fig. 8f, g) and significantly reduced hemorrhage volumes in cases of IA rupture (Fig. 8h) compared to the control mice. These effects did not appear to be due to differences in the size and morphology of aneurysms, as the intima-media thickness (Fig. 8i) and wall-to-lumen ratio (Fig. 8j) were similar in both groups. Interestingly, CAM-depleted mice not only exhibited a significantly lower accumulation of CD206^+^ cells (Fig. 9a–c) but also had significantly fewer neutrophils (Fig. 9d–f), compared to the control (PBS-treated) mice. Indeed, CAMs are in an ideal anatomical position to contribute to focal chemokine gradients, which may either be transported to the luminal surface of endothelial cells and/or act directly as a migratory signal to peripheral immune cells including neutrophils.

## Discussion

While leukocyte, and in particular neutrophil recruitment into the vessel wall, has been extensively studied in both experimental and human IA models [6, 16, 17, 24, 25, 44, 46], the role of tissue resident immune cells such as CAMs remains unexplored in IA formation and rupture. In this study, we aimed to define the role of CAMs in IA pathophysiology, by developing a new experimental model of IAs at the MCA, which represent 20% to 43% of all IAs in humans [21, 22, 42]. Given the spatial localization of a subset of CAMs within the perivascular space (the perivascular macrophages, PVMs), we hypothesized that they could directly or indirectly modulate IA formation, growth and/or rupture. The experimental model developed in this study shares many pathological characteristics found in human MCA IA, including morphological changes and inflammatory cell infiltration. We further demonstrate a pivotal role of CAMs in IA pathophysiology, supported by the reduced IA formation and rupture rates following CAM-specific depletion with intracerebroventricular injection of CLO liposomes prior to IA induction. Moreover, the absence of CAMs ameliorated the severity of IA ruptures resulting in significantly smaller rupture rate and hemorrhage volumes, accompanied by a reduced neutrophil infiltration. Additionally, our in vivo longitudinal molecular MRI studies of VCAM-1 and P-selectin adhesion molecules revealed vascular inflammation in the IA area prior to aneurysm formation. This vascular inflammation correlates with and predicts the severity of bleeding in the case of IA rupture.

In recent years, PVMs have been recognized as an important component of the BBB alongside vascular cells, e.g., endothelial, pericytes, vSMCs, and glia, including microglia, astrocytes, and oligodendrocytes [50]. Under physiological conditions, the BBB restricts leukocyte access to the brain parenchyma; however, BBB disintegration due to inflammatory responses permits entry of leukocytes into the CNS [47]. Our present work aligns with recent studies showing the accumulation of CD206+ macrophages in a murine model of elastase-induced IAs [31] and CD163+ cells in the walls of human IAs [16]. This accumulation is likely a result of in situ proliferation of resident CAMs and/or the infiltration of peripheral macrophages into the IA site.

CAMs have also been shown to mediate neutrophil migration in response to acute ischemic stroke [38]. Our data support the hypothesis that CAMs may directly impact the transmigratory activity of leukocytes, since CLO-induced CAM depletion reduced the accumulation of both neutrophils and CD206^+^ cells in the IA walls. Indeed, PVMs could attract leukocytes, including neutrophils, to inflammation sites by producing chemoattractants such as Cxcl1, Cxcl2, Ccl2, Ccl3, and Ccl4 as described in a model of bacterial skin infection [1]. Moreover, the same study showed that neutrophils preferentially extravasate into the circumscribed locations adjacent to PVMs. In our case, PVMs are in an ideal anatomical position to contribute to focal chemokine gradients [8, 9], which may either be transported to the luminal surface of endothelial cells and/or act as a migratory signal for peripheral immune cells. Data from isolated CAMs in naïve conditions revealed their ability to produce different chemoattractants under physiological conditions, including different interleukins and chemokines [18] involved in neutrophil recruitment [14, 30, 32, 34, 35, 41]. Given that neutrophils are a major source of matrix-degrading proteases, cytotoxic compounds such as elastase and MPOs, and can release NETs [24, 44], CAMs could indirectly mediate IA wall degradation and exacerbate ruptures through neutrophil recruitment, as confirmed by our present data [26]. Consistently, we found that neutrophils in the IA vessel wall were positive for MPO, DNA, and citrullinated histones, indicating that these cells undergo NET formation. NET release correlates with exacerbated vessel wall damage, sustained inflammation through cytokine secretion, and additional leukocyte recruitment—all contributing to IA ruptures [24].

It is worth mentioning that CLO affects not only monocytes/macrophages but also neutrophils when administered intravenously [7]. In our case, CLO was administered intracerebroventricularly 5 days prior to aneurysm surgery. This administration way method allows for the diffusion of clodronate across the closed compartment of the CSF, where it can be taken up by the targeted cells, leading to their selective depletion [39]. As this is a closed compartment where these macrophages reside and no neutrophils are present in healthy conditions, we do not believe that i.c.v. injection of CLO affects the cells from the periphery (in the bloodstream). Also, this approach has been successfully utilized in numerous prior previous studies as a way to selectively deplete CAMs [10, 11, 13].

It is possible that morphological changes in the vessels described here provoke not only an increase in CAM number and their cytokine production, but also a shift in CAM phenotype and function from a scavenger, “buffer” cell to a proinflammatory, ROS-producing cell [4], exacerbating IA ruptures. Oxidative stress is a key contributor to IA development and rupture as it induces direct endothelial injury. Furthermore, free radicals produced by both infiltrating neutrophils and macrophages may induce endothelial dysfunction and further cell recruitment into vessel walls through activation of NF-κB and MCP-1, amplifying the inflammatory response [45]. Faraco et al. [13] have also reported that elevated blood pressure activates ROS production by PVMs through the Angiotensin II type 1 receptor.

The TGF-β signaling pathway plays a crucial role in aortic aneurysms, yet its intrinsic impact remains unclear due to its complexity. Tgfbr2 ablation in smooth muscle cells (SMCs) leads to reduced canonical SMAD signaling, causing stress-related signaling activation and aortic abnormalities. SMC-specific loss of TGF-β signaling, especially in hypercholesterolemic conditions, promotes SMC remodeling into various cell types, contributing to aneurysm development. Mutations in TGF-β pathway genes are associated with aortic aneurysms, and Loeys-Dietz syndrome (LDS) results from dysfunctional TGF-β signaling. Paradoxically, tissues from patients with LDS exhibit enhanced TGF-β signaling. Neutralizing TGF-β increases susceptibility to aneurysms, and anti-TGF-β antibodies lead to AAA development, ruptures, and increased severity. However, complete inhibition of TGF-β may have adverse effects on aortic wall homeostasis, suggesting a delicate balance in therapeutic interventions [5]. CAMs do express TGF-β in naïve conditions (https://anratherlab.shinyapps.io/strokevis/). In our model, CAM depletion leads to a decreased aneurysm formation and rupture rate. Our data suggests a potential role of CAM-derived TGF-β as a triggering factor for aneurysm formation and rupture. However, further investigations are warranted to elucidate the specific contribution of CAM-derived TGF-β in the IA pathophysiology.

The transmigration of leukocytes is mediated by adhesion molecules expressed at the surface of activated endothelial cells [47]. Our results show that specifically at the IA area, endothelial cells express both P-selectin and VCAM-1 at the luminal surface, as evidenced by both immunofluorescence and molecular MRI. Notably, the signal voids obtained by molecular MRI were predictive of IA formation site at early stages, and also of the severity of IA ruptures at later stages. Indeed, we detected a positive correlation between the degree of vascular inflammation and the post-rupture hemorrhage volumes. Our results indicate that molecular MRI of vascular inflammation holds promise for future IA prognosis and treatment management. Our study proposes that CAMs are one of the main actors orchestrating the formation and rupture of IAs in our IA mouse model. However, further studies are required to determine whether this pathological role is primary or secondary, possibly through the modulation of neutrophil recruitment and/or microglial activation, rather than CAMs acting alone. Our data contribute to a much-needed improvement of the management and understanding of clinical IA progression and the inflammatory pathways involved in IA rupture. Considering their promoting and exacerbating effects on IA ruptures, CAMs may be valid targets for potential novel therapeutic strategies in IA pathogenesis. Furthermore, our molecular MRI data suggest that this imaging tool may hold diagnostic and prognostic value for future clinical IA treatment and management.

Our model, despite its effectiveness in mimicking human IA pathophysiology with morphological and inflammatory changes, has certain limitations. It is important to keep in mind that in vivo animal models of IAs, including our IA MCA model, do not fully replicate the protracted and multifactorial nature of human IA development. The necessity to artificially accentuate inciting factors (e.g., Ang II infusion and elastase injection), is a practical measure to achieve a severe disease phenotype within a constrained, feasible timeframe. However, this intentional accentuation may not entirely capture the gradual and accumulative nature of IA dynamics in humans, where persistent chronic inflammation plays a crucial role. Moreover, these physiological insults employed to induce our model, particularly hemodynamic stress and elastase administration, could trigger inflammatory processes by activating endothelial cells. While this is a crucial aspect of our experimental design, it is important to recognize that the inflammatory response initiated by induction of IAs in our model may not perfectly mirror the complex and nuanced chronic inflammation seen in human aneurysm progression. Additionally, aneurysm formation, growth, and rupture in humans typically unfold over an extended period, contrasting with the more accelerated timeline necessitated by experimental constraints. In light of these limitations, careful consideration is warranted when extrapolating our findings to the clinical context, emphasizing the need for further studies that explore the intricate and dynamic nature of IA pathogenesis in humans.

## Methods and materials

### Induction of aneurysms in MCA IA model

Formation of IAs was induced by local microinjection of elastase (1µL, 35 IU) behind the MCA bifurcation (Fig. 1a; Additional file 1: Figure 1a) and immediately followed by subcutaneous Angiotensin II (Ang II) osmotic pump (800 µg in 90 µL of saline solution; diffusion rate 37 ng/min/14 days) insertion (Fig. 1a; Additional file 1: Figure 1a). This model is a variation of the model previously described by Nuki et al. [37], inspired by models developed for abdominal aortic aneurysm studies [2]. The dose of elastase was chosen after a dose–response test where different parameters were studied (i.e., IA formation, occurrence of hemorrhage, survival rate etc.) (Additional file 1: Figure 2a).

To avoid including animals with potential surgically induced hemorrhages in the study, all animals were thoroughly assessed 24 h post-surgery by using the specific T2*-w sequence to visualize potential hemorrhages. Mice presenting spontaneous postoperative hemorrhages were excluded from further studies (Fig. 1a). Signal void appearing at the later stages (after aneurysm development) in T2*-w were considered aneurysm ruptures (Additional file 1: Figure 1e–h). To confirm that the vessel wall degradation was caused by the elastase micro-injection and was not due to potential micropipette-induced vascular damage, a control experiment was performed by injecting PBS (n = 5) or elastase (35 mU/µL) (n = 8). None of the PBS-injected mice developed aneurysms, and no hemorrhage was observed at any time throughout the protocol (Additional file 1: Figure 1i).

### Human SCAA sample

The studied aneurysm sample was resected by Dr. Frösen at the Kuopio University Hospital, following clipping of the aneurysm neck. The sample was from a large middle cerebral artery aneurysm with a diameter of 2cm. A summary of the preoperative imaging studies is presented in Fig. 3. An informed consent was obtained from the patient prior to the procedure, and the study was approved by the Ethical committee of the Hospital District of Northern Savo (TKU 52 2014). Following resection, the tissue sample was fixed overnight in 4% paraformaldehyde and embedded in paraffin so that the original shape of the aneurysm dome was preserved.

### Histology and immunofluorescence staining of human samples

The resected human MCA aneurysm sample was cut into 4 µm thick sections with a microtome, following which these sections were deparaffinized and rehydrated using a standard alcohol series. After rehydration, some of the sections were stained with standard hematoxylin–eosin staining. For those sections that were immunostained, an antigen retrieval procedure was performed by incubating the sections in close to boiling temperature in HIER buffer (ThermoFisher Scientific, Watham, MA, USA) for 2 × 5min. After that, a protein block in 3% normal horse serum (Biowest, Nuaillé, France) in 0.1% PBST for 30 min at room temperature was performed. Following the antigen retrieval and serum block, the sections were incubated overnight at 4 °C with the primary antibody (Table 1) diluted in 1.5% normal horse serum (Biowest) in 0.1% PBST. Following this, after 3 × 5 min washes in PBS, the sections were incubated with the secondary antibody (Vector Laboratories, Burlingame, CA) (dilution 1:200) in 1.5% normal horse serum in 0.1% PBST for 30 min at room temperature. Secondary antibodies were chosen according to the species where primary antibodies were produced. After the incubation with the secondary antibody, the sections were washed for 3 × 5 min in PBS and underwent an endogenous peroxidase block with 3% hydrogen peroxide (ThermoFisher Scientific) in PBS for 20 min at room temperature. This was followed by 3 × 5 min PBS wash, following which the sections were incubated with a horseradish peroxidase conjugated avidin–biotin complex (Vector Laboratories) for 30min in room temperature. After subsequent 3 × 5min PBS washes, the positive signal was detected with DAB (3′–5′-diaminobenzidine) (Vector Laboratories). Hematoxylin was used for counter staining and the stained sections dehydrated and mounted with Depex.

**Table 1:** Primary antibodies used for histology and immunofluorescence

Markers	Detection	Species	Dilution	Manufacturer	Cat. number
*Mouse samples*					
α-SMA	α-smooth muscle actin	Mouse	1:500	Abcam	ab8211
CD206	CAMs	Rat	1:500	BioRad	MCA2235GA
CD3	T-lymphocytes	Rabbit	1:200	Abcam	ab5690
CD31	Endothelial cells	Rabbit	1:500	Abcam	ab28364
CD62P	P-selectin	Rat	1:500	BP Pharmingen	553742
CD68	Lysosomes	Rat	1:500	Abcam	ab53444
Col IV	Blood vessels	Goat	1:1000	SouthernBiotech	1340-01
Fibrinogen	Fibrinogen	Goat	1:1000	Non-commercial	
H3Cit	Neutrophil extracellular traps	Rabbit	1:500	Abcam	ab5103
Iba1	Microglia	Goat	1:1000	Abcam	ab5076
Laminin	Blood vessels	Rabbit	1:1500	Abcam	ab11575
Ly6G	Neutrophils	Rat	1:500	StemCell	60031
MPO	Myeloperoxidase	Rabbit	1:500	Abcam	ab9535
P2Y12R	Microglia	Rabbit	1:500	Anaspec Inc	as55043A
*Human samples*					
CD68	Lysosomes	Mouse	1:500	Dako	M0814
MHCII	MHCII molecules	Mouse	1:500	Santa Cruz	sc-53302
CD11b	Monocytes/macrophages, granulocytes, natural killer cells	Mouse	1:50	Santa Cruz	sc-1186
CD163	CAMs	Rabbit	1:50	Novusbio	NBP3-08325B

Double immunofluorescence staining was performed to confirm the co-localization of markers CD11b and CD163. Protocol was similar to the one described above, except that normal goat serum (Vector Laboratories) in 0.1% PBST was used for protein blocking and antibody dilutions. Secondary antibodies were AlexaFluor 488 and 594 conjugated (dilution 1:200, ThermoFisher Scientific) for signal detection. Mounting was performed with Vectashield medium with DAPI (Vector Laboratories).

The sections chosen for the staining of human samples were adjacent sections from different depths of the IA, making it possible to compare co-localization of different inflammation markers. Immunoperoxidase stained sections were imaged with Olympus AX70 microscope (Olympus, Japan). For immunofluorescence staining, imaging was performed with LSM800 Zeiss confocal microscope system (Carl Zeiss Ag, Oberkochen, Germany) with 405/488/555 nm diode lasers and appropriate emission filters (20x/0.5 PlanApo objectives, 1024 × 1024 frame sizes). Image processing was performed by ImageJ (Rasband, W.S., National Institutes of Health, Bethesda, Maryland).

### Animals

8 weeks old male Swiss (Janvier Labs) mice were housed (Centre Universitaire de Ressources Biologiques, Normandy University, Caen, France) at 21 °C in a 12 h light/dark cycle with food and water ad libitum. Animal care and manipulations complied with recommendations issued by the French and European guidelines for the care and use of laboratory animals (European directive 2010/63/UE) and were authorized by the ethical committee (authorization no. 27499). All experiments were performed following the ARRIVE guidelines (www.nc3rs.org.uk). All the procedures needing anesthesia were performed by an initial exposure to 5% isoflurane followed by a maintaining phase of 1.5–2% isoflurane 30% O2/70% N_2_O during experiments.

### Depletion of CAMs

Depletion of CAMs was carried out 5 days before the MCA aneurysm induction surgery to minimize the pro-inflammatory effects of CLO per se, by injecting CLO intracerebroventriculary. Anesthetized mice were placed in a stereotaxic device, and after the skin was removed, a small craniotomy was performed (coordinates: − 0.2 mm anteroposterior; + 1 mm lateral; − 2 mm depth from the Bregma). With a glass micropipette, 10 µl PBS-liposomes (PBS group) or clodronate-encapsulated liposomes (CLO group) (purchased from clodronateliposomes.com) were gently injected into the left lateral ventricle for 20 min. CLO liposomes injected into the cerebral ventricles are transported by the cerebrospinal fluid to the perivascular spaces and phagocytized by CAMs. In the cytosol, CLO acts as a cytotoxic ATP analog, which impairs mitochondrial oxygen consumption and leads to cell death by apoptosis [29].

### Blood pressure measurements

Blood pressure in mice was measured using the Visitech BP-2000 blood pressure analysis system to ensure accurate physiological data while minimizing stress on the animals. Measurements, including arterial blood pressure (systolic, diastolic, mean) and heart rate, were taken every third day using a volume pressure recording sensor and an occlusion tail-cuff system. The experimental setup involved activating the BP-2000 computer and control unit, setting the heating platform to 34 °C, and placing mice in a magnetic restraining device with their tails free. Mouse was gently placed into an integrated animal holder to ensure proper restraint during the procedure. The tail was inserted through the corresponding tail-cuff, taped down, and covered with an LED light sensor. During the analysis cycle, the blood pressure control unit pressurized and attempted to read a pulse, producing results at the end of each measurement cycle. Recording sessions, integrated with data acquisition, were repeated until experimental variability was minimized, typically requiring three sessions.

### Tissue harvesting and fixation of mouse samples

Brain samples for IHC were isolated 5 and 15 days after the MCA aneurysm surgery. Terminally anesthetized mice were transcardially perfused with cold heparinized saline (15 mL/min) and fixed with 50 mL of 4% paraformaldehyde phosphate buffer (pH 7.4). Brains were post-fixed with 4% paraformaldehyde phosphate buffer (18 h; 4 °C) and cryoprotected (sucrose 20% in PBS; 24 h; 4 °C) before freezing in CryomatrixTM medium, frozen in isopentane cooled with liquid nitrogen, and stored at − 80 °C.

### Immunofluorescence (mouse samples)

For IF analysis 10 μm thick cryostat-cut sections were adhered and collected on poly-lysine coated microscope slides and stored at − 80 °C before processing. Sections were randomly selected and incubated overnight at room temperature, with primary antibodies listed in Table 1. Primary antibody solutions were prepared with PBS-Triton (25% v/v). To reveal primary antibodies, after rinsing three times in PBS (1x, pH = 7.4), brain sections were incubated with appropriate Donkey Fab’2 fragments conjugated with Alexa Fluor® 488, Alexa Fluor® 594 or Alexa Fluor® 647 (1:1000, Jackson ImmunoResearch, West Grove, USA).

After another set of rinsing in PBS (1x, pH = 7.4) and deionized water, slides were left to dry. Finally, sections were coverslipped with an antifade medium containing DAPI. Images were captured using Leica DM6000 epifluorescence microscope coupled camera and visualized with Leica MM AF 2.2.0. software (Molecular Devices, USA). Additionally, confocal images were taken on Leica TCS SP5 MP microscope using the 40X oil immersive objectives. Images were taken using LAS AF Leica Software.

### Automated microglial morphology analysis

Following transcardial perfusion with 4% PFA, 100 µm thick brain sections were cut with vibratome and stained for microglia (Iba1, Ancam Ab5076) and cell nuclei (DAPI, Sigma 10236276001). Confocal stacks with 0.2 µm/pixel size and Z-step of 0.4 µm were acquired using Nikon A1R confocal microscope with 60 × water immersion objective (Plan Apo VC NA = 1.2 WD = 0.31–0.28 mm FOV = 215.04 µm; Nikon Instruments Europe B. V., Amsterdam, the Netherlands). Images were acquired both on the ipsi- and contralateral side of the aneurysm. Stacks were then analysed using the Microglia Morphology Quantification Tool [20]. This automated analysis provides an unbiased, 3D analysis of microglia morphology.

### Magnetic resonance imaging

Imaging was performed on a Pharmascan 7T/12 cm system using surface coils (Bruker, Germany). Mice were deeply anesthetized with 5% isoflurane and maintained with 1.5–2% isoflurane 30%O_2_/70%N_2_O during the acquisitions. T2-weighted images were acquired using a multislice sequence: TE/TR 33/2500 ms. Hemorrhage volumes were quantified using the ITK software. Mice showing lesions < 6mm3 at 24 h post-surgery were considered as surgical failure and excluded from the analyses.

For molecular MRI (see below) three-dimensional T2*-weighted gradient echo imaging with flow compensation (field-of-view (FOV) 70 × 70 × 70 mm^3^ interpolated to an isotropic resolution of 70 mm), TE/TR 13.2 ms/200 ms and a flip angle of 21° was performed to visualize MPIOs. T2*-weighted sequences were used to verify if IAs ruptured.

### Molecular MR Imaging of vascular inflammation and quantification

Micro-sized particles of iron oxide (MPIOs) (1.08 μm diameter; Dynabeads MyOne Tosyl Activated, Invitrogen) covalently conjugated to purified polyclonal goat anti-mouse antibodies for P-selectin (R&D Systems, clone AF737) and purified polyclonal mouse anti-rat antibodies for VCAM-1 (Clone MR106, BD Pharmingen) were prepared as previously described [32]. In brief, MPIOs were covalently conjugated to polyclonal antibodies in borate buffer (pH 9.5) at 37 °C for 48 h. The coating of the beads was made using 20 μg of VCAM-1 or 40 μg of P-selectin antibody per 1 mg of reactive MPIOs. After, beads were washed and incubated with PBS containing 0.5% BSA for 24 h at room temperature to block remaining active groups. MPIO solution is stored under continuous rolling at 4 °C until usage.

MRI acquisitions started immediately after the intravenous injection of MPIO-solution (200 µl of 2 mg Fe/kg of conjugated MPIOs). All T2*-weighted images presented are minimum intensity projections of six consecutive slices. The qualitative analysis and the distribution of the MPIO signal voids on 3D T2*-weighted images was assessed by ITK Snap Software [49].

Semi-automatic signal void quantification was performed similar to previously described [15]. Briefly, signal voids corresponding to MPIO presence were quantified in subject space within region-of-interest (ROI) masks of the aneurysm lesion and the ipsilateral hemisphere and their contralateral homologues. In MATLAB, histograms of voxel signal intensities in each T2*-weighted image in contralateral ROIs were generated and fitted to a Gaussian of certain peak center and peak width. Voxels in the brain with signal intensities lower than the value at the contralateral peak center minus its half width (i.e., signal intensity values lower than 6.25% of the value of the majority of contralateral reference voxels) were subsequently labeled as signal void. Per time point, signal voids were counted per ROI and expressed as a percent-difference of the count in the corresponding contralateral ROI.

### Flow cytometry

After transcardiac perfusion with PBS, brains were roughly minced and homogenized with a potter tissue grinder in Hanks’ balanced salt solution (HBSS) containing 15 mM HEPES buffer and 0.54% glucose. Whole brain homogenate was separated by 37% Percoll gradient centrifugation at 800*g* for 30 min at 4 °C (no brake). The pellet containing CNS leukocytes at the bottom of the tube was then collected and washed once with PBS containing 2% FCS before staining.

Fc receptors were blocked with CD16/32 (553142, BD Biosciences) for 10 min at 4 °C before incubation with the primary antibodies. Cells were stained with antibodies directed against CD11b (M1/70, BioLegend), CD45 (30-F11, BD Biosciences), Ly6G (1A8, BD Biosciences), CD3e (145-2C11, BD Biosciences) and CD206 (C068C2, BioLegend) for 45 min at 4 °C. After washing, samples were analyzed by a FACSVerse flow cytometer or sorted by a FACSAria (BD Biosciences). Appropriate isotype control antibodies were used to establish sorting parameters. Data were analyzed with the FlowJo 7.6.5 software (TreeStar Inc.). Data are expressed as percentages.

### Elastica van Gieson staining

For histological examination of brain tissue sections and visualization of connective tissue made up of elastic fibers Elastica van Gieson staining kit was used (Merck, Darmstadt, Germany). Slides with 10 μm thick brain sections were incubated for 13 min in resorcin fuchsin solution and then for 5 min in alcoholic hematoxylin solution and hydrochloric acid iron(III)nitrate solution in ratio 1:1. After each incubation slides were rinsed under running tap water for 1 min. Slides were then incubated in pirofuchsin solution (picric acid/acid fuchsin solution) for 2 min and rinsed in 70%, 96% and 100% ethanol (two times for 1 min in each percentage). Finally, slides were incubated in toluene for 10 min and coverslipped with mounting medium. Images were captured using Leica DM6000 epifluorescence microscope coupled camera and visualized with LAS X Leica Software (Molecular Devices, USA).

### Measurements of the vessels and cell quantification

All images were analyzed using ImageJ 1.51k software. To determine the intima-media thickness and wall-to-lumen ratio, first, the diameters of both vessel and vessel lumen were measured. Intima-media thickness was calculated as a half value of the vessel diameter subtracted by the diameter of the lumen. The results are expressed in μm. To calculate wall-to-lumen ratio, the value of intima-media thickness was divided by the lumen diameter.

CD206^+^, Ly6G^+^, and CD3^+^ cells were quantified in a nonrestricted area around the aneurysms in the ipsilateral hemisphere and control arteries in the contralateral hemisphere. DAPI staining for cell nuclei was used to confirm that signal comes from the cell. Results for each cell type are presented as a number of cells around the aneurysm and contralateral artery. Microglial activation and proliferation were analyzed on images taken using 20X objective of Leica DM6000 epifluorescence microscope coupled camera, in an area of 400 × 400 μm. All quantifications were done manually, blinded to the treatment.

### Image visualization

Images presented in the in Fig. 1; Figs. 2 and 3 and Additional file 1: Fig. 1 were adapted from BioRender with postprocessing using PowerPoint.

### Statistical analysis

All data are expressed as the mean ± standard error from the mean (s.e.m.) with the analysis being conducted using two-way ANOVA with Dunnett’s post hoc multiple-comparisons or Mann–Whitney U test. Two-Way ANOVA was used to compare the values between ipsi- and contralateral hemispheres in CLO and PBS-treated groups. Fisher’s exact test was used to compare the aneurysm rupture rate between the PBS and CLO treated groups. The Mann–Whitney U test was used for the comparison between two groups. Significance was set at *P* < 0.05. N values refer to the number of replicates. All statistical analysis and plotting were performed in GraphPad 9 (GraphPad). All quantifications were done manually, blinded to the treatment.

### Supplementary Information

**Additional file 1: Fig. S1.** Study design and MCA model characterization **a**; The IA model was designed and realized by injection of elastase in young-adult Swiss male mice combined with a subcutaneous infusion of Angiotensin II over 14 days (Osmotic pump p1001, Alzet®). 24h after the surgery, T2*-w MR scan was performed to exclude the animals with substantial bleedings due to the surgery. First, the cranial window was opened, and the MCA bifurcation was found. Elastase (1µL, 35IU) was injected behind the MCA bifurcation, thereafter. Hypertension was induced by the Ang II osmotic pump. This led to hemodynamical changes and clear morphological alterations in the MCA, promoting the appearance of IAs. **b**; Mean arterial pressure (MAP) in mice that received Ang-II is increased in compared with the mice with no pump inserted. **c**; A dose-dependent relationship between the animals that received Ang-II and the animals with no pump inserted. (***p<0.001, n=8 per group, Mann-Whitney U test per each time-point) **d**; Example of hemorrhages in our MCA IA model – intraparenchymal and subarachnoid hemorrhage (Day 10). **e**; Assessment of the IA ruptures. IAs are visible using the T1-w sequence with gadolinium as a contrast agent, whereas the rupture is assessed by a T2*-w sequence. After the rupture, the IA is no longer visible at the T1-w MR scan. **f**; Development of the IA with time on the MRI. e; Representative T1-w MR scans with gadolinium as a contrast agent of IA at the outer part of the brain in coronal (on the left) and axial (on the right) orthogonal projection. **g**; MR scans used to detect IAs in the histological slices found typically at the outer part of the brain. **h**; Rupture of IA results in a hyposignal visible with the T2*-w MR sequence. **i**; Neither IAs nor hemorrhages are observed in PBS injected mice (n=5) during the 15-day study period. **Fig. S2.** Dose-dependent elastase effect and MR scan assessment **a**; Dose-dependent effects of elastase on the incidence of IAs and the hemorrhagic transformations. Three different concentrations of elastase were tested, plus the control (n=27). A control group was injected with the Saline 1µL. Different concentrations of the elastase; 17mU/μL; 35mU/μL and 70mU/μL. On the right, percentage of deaths among the groups assessed at the end of the protocol. IA repartition and hemorrhage occurrence compared at different time-points among different groups. Control group showed no hemorrhages 24h post-surgery, and no deaths. Data was assessed blindly. **b**; The occurrence of IAs at different time points reveal that mice without inserted pumps had fewer developed aneurysms and fewer ruptures as well. **c**; The average number of IAs per animal quantified from T1-w MRI during the 15-day timeline shows a smaller number of developed aneurysms per animal, mostly one per animal if the animal developed an IA (n=9; 4/9 did not develop aneurysms). **d**; Mice without the pumps exhibited a decreased global rupture rate, although statistically nonsignificant (ns, n=14 control mice [with pump]/n=9 without the pump; With versus Without the pump: 72% versus 60%, Fisher exact test). Data was assessed blindly. **e**; MRI scans used to detect the IAs in the histological slices found typically at the outer part of the brain. **Fig. S3.** Molecular MRI of vascular inflammation: study design and quantification approach **a**; Schematic representation of molecular MRI. The technique is based on microparticles of iron oxide (MPIOs) used as a contrast agent for the MRI. MPIOs are coupled with large number of the antibodies on their surface for cell adhesion molecule of our interest (VCAM-1 and P-selectin). Once injected in the blood system, the antibodies at the surface of the particles specifically target adhesion molecules expressed at the surface of activated cerebral endothelial cells, which results in signal assessed by the MRI. MPIOs are restricted to the vascular compartment and cannot passively extravasate into the brain parenchyma. **b**; Semi-automatic quantification approach for the molecular MRI; Signal from the 3D-T2*-w MR scan was calculated with both ITK-Snap and MatLAB (described in detail in Material and Methods section). Two corrections were done, one for the pre-injection image and the potential signal noise, and the other for the contralateral side which was considered as a control side. **Fig. S4.** Molecular MRI: P-Selectin and VCAM-1 early signal assessment. An early timepoint of P-selectin and VCAM-1 expression (Day3) is shown. Representative MRI images before and after intravenous injection of MPIO-α-P-selectin and MPIO-αVCAM-1 at the Day3 following the IA formation.

## Abbreviations

α-SMA: α-Smooth muscle actin
ATP: Adenosine triphosphate
BBB: Blood brain barrier
CAMs: CNS-associated macrophages
ChPM: Choroid plexus macrophages
Col IV: Collagen type IV
CLO: Clodronate
CNS: Central nervous system
EC: Endothelial cell
FACS: Fluorescence-activated cell sorting
H3Cit: Citrullinated histone H3
IAs: Intracerebral Aneurysms
Iba1: Ionized calcium-binding adapter molecule-1
i.c.v.: Intracerebroventricular
IEL: Internal elastic lamina
IF: Immunoflourescence
Ly6G: Lymphocyte antigen 6 complex locus G
MCA: Middle cerebral artery
MCP-1: Monocyte chemoattractant protein-1
MHCII: Major histocompatibility complex class II
MM: Meningeal macrophages
MPO: Myeloperoxidase
MPIOs: Microparticles of iron oxide
MRI: Magnetic resonance imaging
NETs: Neutrophil extracellular traps
NF-κB: Nuclear factor-κB
PBS: Phosphate-buffered saline
PECAM-1: Platelet endothelial cell adhesion molecule
Psel: P-selectin
PVMs: Perivascular macrophages
ROS: Reactive oxygen species
SAH: Subarachnoid hemorrhage
VCAM-1: Vascular cell adhesion molecule-1
