# Supplementary material for: CNS-associated macrophages contribute to intracerebral aneurysm pathophysiology
Source: Acta Neuropathol Commun. 2024 Mar 18;12:43. doi: 10.1186/s40478-024-01756-5 (PMC10946177; doi:10.1186/s40478-024-01756-5)
Supplement: Supplementary file 1 — Additional file 1: Fig. S1. Study design and MCA model characterization a; The IA model was designed and realized by injection of elastase in young-adult Swiss male mice combined with a subcutaneous infusion of Angiotensin II over 14 days (Osmotic pump p1001, Alzet®). 24h after the surgery, T2*-w MR scan was performed to exclude the animals with substantial bleedings due to the surgery. First, the cranial window was opened, and the MCA bifurcation was found. Elastase (1µL, 35IU) was injected behind the MCA bifurcation, thereafter. Hypertension was induced by the Ang II osmotic pump. This led to hemodynamical changes and clear morphological alterations in the MCA, promoting the appearance of IAs. b; Mean arterial pressure (MAP) in mice that received Ang-II is increased in compared with the mice with no pump inserted. c; A dose-dependent relationship between the animals that received Ang-II and the animals with no pump inserted. (***p<0.001, n=8 per group, Mann-Whitney U test per each time-point) d; Example of hemorrhages in our MCA IA model – intraparenchymal and subarachnoid hemorrhage (Day 10). e; Assessment of the IA ruptures. IAs are visible using the T1-w sequence with gadolinium as a contrast agent, whereas the rupture is assessed by a T2*-w sequence. After the rupture, the IA is no longer visible at the T1-w MR scan. f; Development of the IA with time on the MRI. e; Representative T1-w MR scans with gadolinium as a contrast agent of IA at the outer part of the brain in coronal (on the left) and axial (on the right) orthogonal projection. g; MR scans used to detect IAs in the histological slices found typically at the outer part of the brain. h; Rupture of IA results in a hyposignal visible with the T2*-w MR sequence. i; Neither IAs nor hemorrhages are observed in PBS injected mice (n=5) during the 15-day study period. Fig. S2. Dose-dependent elastase effect and MR scan assessment a; Dose-dependent effects of elastase on the incidence of IAs and the hemorrhagic [file 40478_2024_1756_MOESM1_ESM.pdf]

Supplementary Material

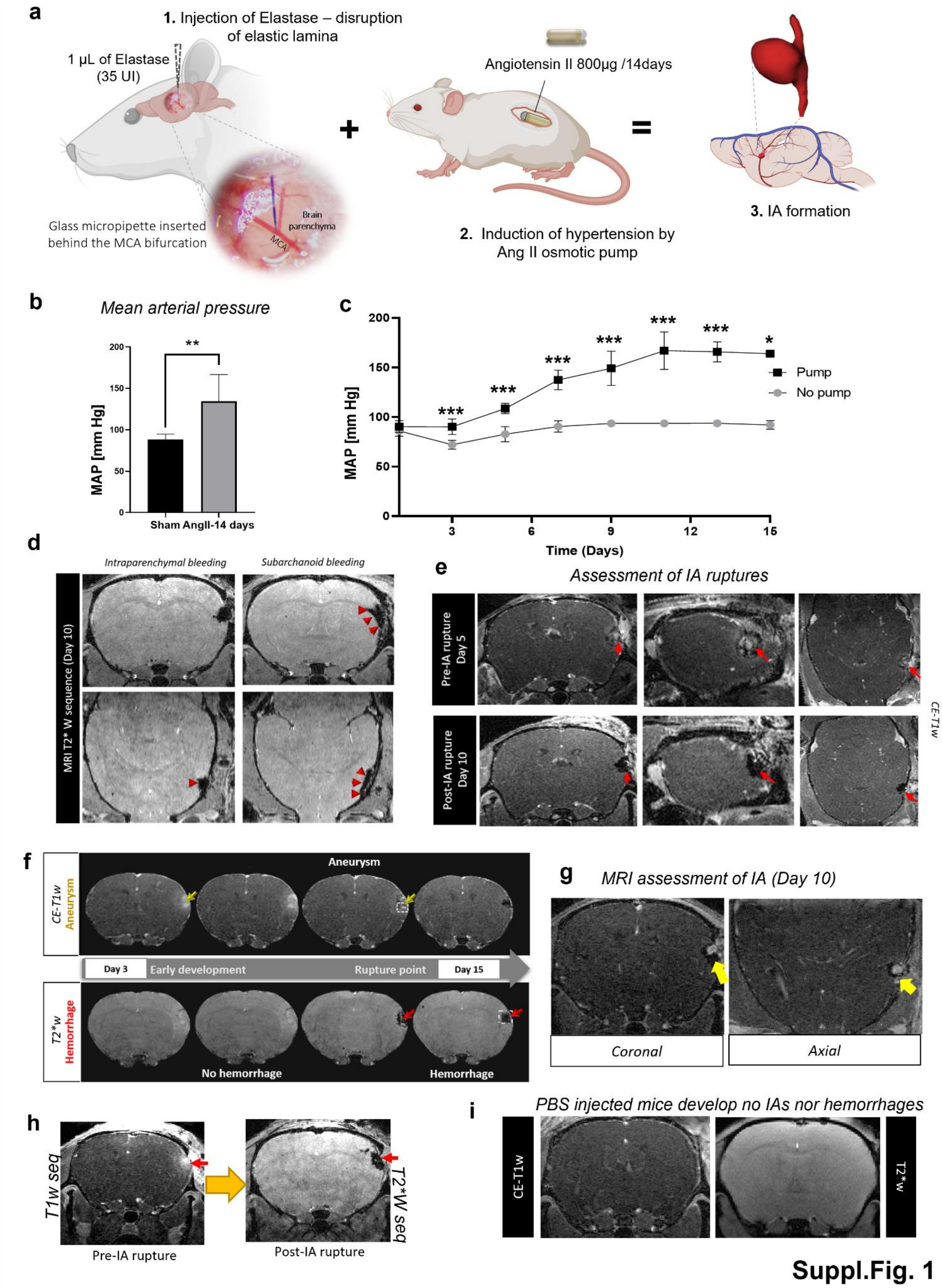

Suppl.Fig. 1

CNS-associated macrophages contribute to intracerebral aneurysm pathophysiology, *Acta Neuropathol. Commun.*, Glavan et al. (corresponding author: rubio@cyceron.fr)

Supplementary Material

Suppl. Fig.1 Study design and MCA model characterization a; The IA model was designed and realized by injection of elastase in young-adult Swiss male mice combined with a subcutaneous infusion of Angiotensin II over 14 days (Osmotic pump p1001, Alzet®). 24h after the surgery, an MRI scan (T2*-w) was performed to exclude the animals with substantial bleedings due to the surgery. First, the cranial window was opened, and the MCA bifurcation was found. Elastase (1µL, 35IU) was injected behind the MCA bifurcation, thereafter. Hypertension was induced by the Ang II osmotic pump. This led to hemodynamical changes and clear morphological alterations in the MCA, promoting the appearance of IAs. **b;** Mean arterial pressure (MAP) in mice that received Angiotensin-II is increased in compared with the mice with no pump inserted. **c;** A dose-dependent relationship between the animals that received Angiotensin-II to, and the animals with no pump inserted. (***p<0.001, n=8 per group, Mann-Whitney U test per each time-point) **d;** Example of hemorrhages in our MCA Aneurysm model – intraparenchymal and subarachnoid hemorrhage (Day 10). **e;** Assessment of the IA ruptures. IAs are visible using the T1-w sequence with gadolinium as a contrast agent, whereas the rupture is assessed by a T2*-w sequence. After the rupture, the IA is no longer visible at the T1-w MR scan. **f;** Development of the IA with time on the MRI. **e;** Representative T1-w MR scans with gadolinium as a contrast agent of IA at the outer part of the brain in coronal (on the left) and axial (on the right) orthogonal projection. **g;** MRI scans used to detect the IAs in the histological slices found typically at the outer part of the brain. **h;** Rupture of IA results in a hyposignal visible with the T2*W MR sequence. **i;** Neither IAs nor hemorrhages are observed in PBS injected mice (n=5) during the 15-day study period.

Supplementary Material

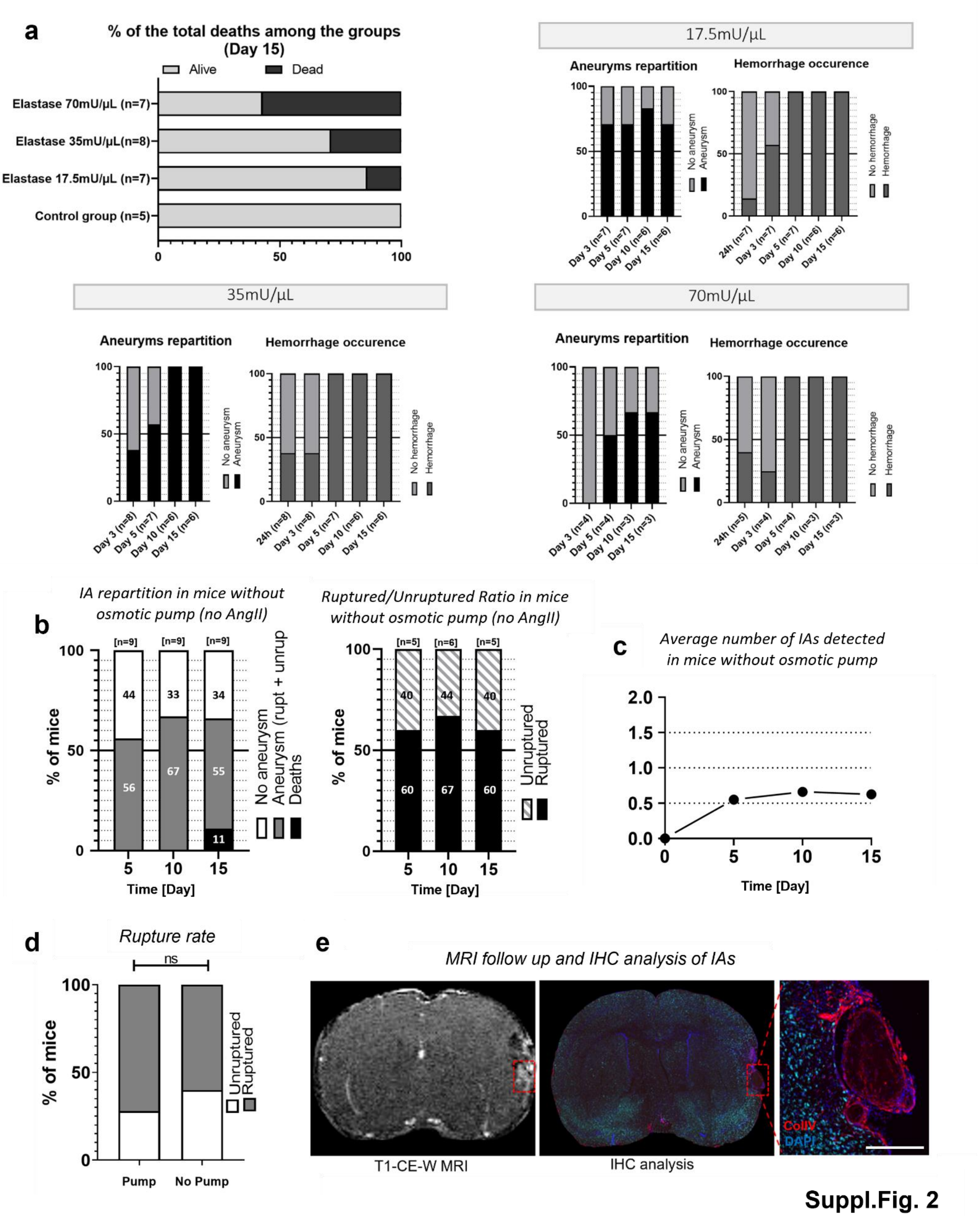

Suppl.Fig. 2

Supplementary Material

Suppl. Fig. 2 Dose-dependent elastase and osmotic pump effect on aneurysm formation and rupture a; Dose-dependent effects of elastase on the incidence of IAs and the hemorrhagic transformations. Three different concentrations of elastase were tested, plus the control (n=27). A control group was injected with the Saline 1µL. Different concentrations of the elastase; 17mU/µL; 35mU/µL and 70mU/µL. On the right, percentage of deaths among the groups assessed at the end of the protocol. IA repartition and hemorrhage occurrence compared at different time-points among different groups. Control group showed no hemorrhages 24h post-surgery, and no deaths. Results were assessed blindly. **b;** The occurrence of IAs at different time points reveal that mice without inserted pumps had fewer developed aneurysms and fewer ruptures as well. **c;** The average number of IAs per animal quantified from T1-w MRI during the 15-day timeline shows a smaller number of developed aneurysms per animal, mostly one per animal if the animal developed an IA (n=9; 4/9 did not develop aneurysms). **d;** Mice without the pumps exhibited a decreased global rupture rate, although statistically nonsignificant (ns, n=14 control mice [with pump] versus n=9 [without the pump]; with versus without the pump: 72% versus 60%, Fisher exact test). **e;** MRI scans used to detect the IAs in the histological slices found typically at the outer part of the brain. Scale bar 500µm.

Glavan et al. (corresponding author: rubio@cyceron.fr)

Supplementary Material

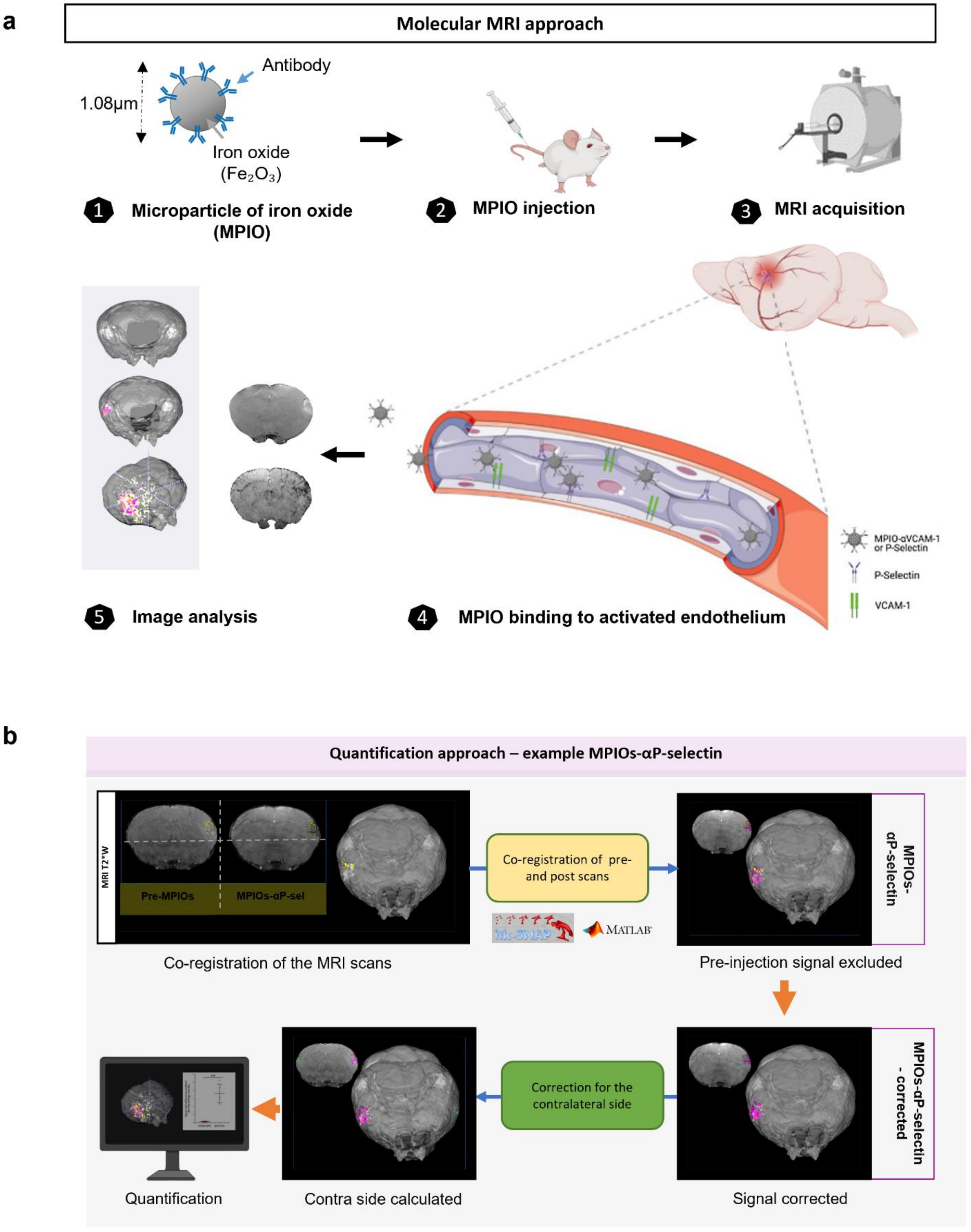

Suppl.Fig. 3

Supplementary Material

Suppl. Fig. 3 Molecular MRI of vascular inflammation: study design and quantification approach a; Schematic representation of molecular MRI. The technique is based on microparticles of iron oxide (MPIOs) used as a contrast agent for the MRI. MPIOs are coupled with large number of the antibodies on their surface for cell adhesion molecule of our interest (VCAM-1 or P-selectin). Once injected in the blood system, the antibodies at the surface of the particles specifically target adhesion molecules expressed at the surface of activated cerebral endothelial cells, which results in signal assessed by the MRI. MPIOs are restricted to the vascular compartment and cannot passively extravasate into the brain parenchyma. **b;** Semi-automatic quantification approach for the molecular MRI; Signal from the 3D-T2*-W MRI was calculated with both ITK-Snap and MatLAB (described in detail in Material and Methods section). Two corrections were done, one for the pre-injection image and the potential signal noise, and the other for the contralateral side which was considered as a control side.

Supplementary Material

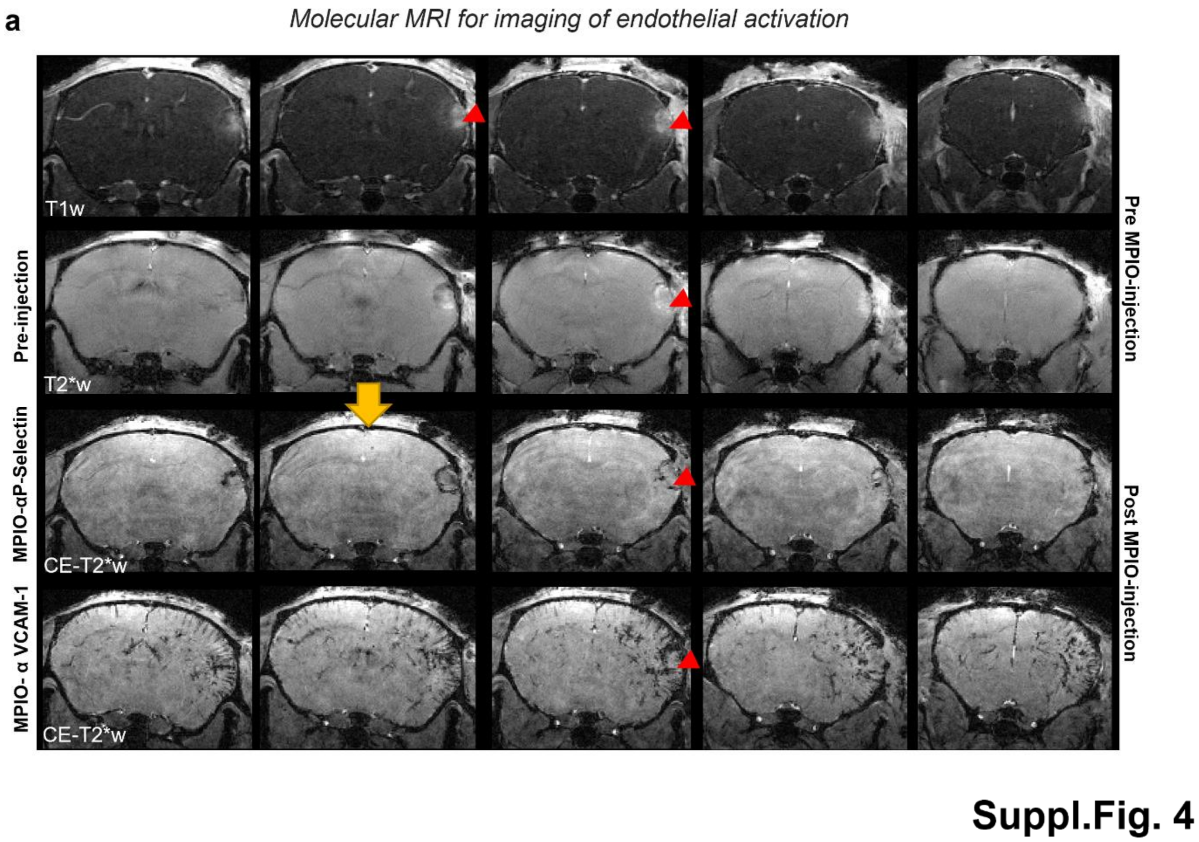

Suppl. Fig. 4 Molecular MRI: P-Selectin and VCAM-1 early signal assessment An early timepoint of P-selectin and VCAM-1 expression (Day3) is shown. Representative MRI images before and after intravenous injection of MPIO-α-P-selectin and MPIO-αVCAM-1 at the Day3 following the IA formation.
